# Non-Monotonic Mechanical Response and Multiscale Damage Evolution of Argillaceous Siltstone Under Wet–Dry Cycles

**DOI:** 10.3390/ma19173678

**Published:** 2026-08-29

**Authors:** Zihang He, Dajin Zhang, Guangli Xu, Neng Zhang, Hankang Zhang

**Affiliations:** 1 Faculty of Engineering, China University of Geosciences (Wuhan), Wuhan 430074, China; 1202420589@cug.edu.cn (Z.H.);; 2 Zhejiang Zhezhong Geological Engineering Investigation Institute Co., Ltd., Jinhua 321000, China

**Keywords:** argillaceous siltstone, wet–dry cycles, water–rock interaction, energy dissipation, digital image correlation, face porosity, weak-rock slopes, particle redistribution

## Abstract

Argillaceous siltstone forms water-sensitive weak layers in red-bed slopes, but its multiscale deterioration under wet–dry cycling remains incompletely understood. Specimens subjected to 0, 1, 3, 5, 7, and 9 cycles were examined through uniaxial compression, energy analysis, digital image correlation (DIC), and microstructural and mineralogical characterization. The mean unconfined compressive strength (UCS) decreased by 36.0% after the first cycle and then remained broadly stable, with modest fluctuations, from 1 to 5 cycles. At five cycles, the elastic modulus remained substantially below the natural-state value, and although total strain energy approached the natural-state level, elastic strain energy remained lower and the dissipated energy ratio more than doubled, indicating continued irreversible damage. The characteristic calcite diffraction peak was no longer detected in the X-ray diffraction (XRD) patterns, while microstructural observations showed redistributed fines within pores together with a temporary decrease in face porosity. With further cycling, the UCS declined again and was 59.0% below its initial level after nine cycles. Meanwhile, strain fields and failure patterns evolved from localized deformation and splitting to distributed cracking and surface spalling, while particle detachment reopened pores and increased face porosity to 14.37%. These observations are consistent with a dissolution–filling–detachment mechanism and suggest that the intermediate UCS stabilization reflected temporary maintenance of load-bearing capacity rather than recovery of the original rock skeleton.

## 1. Introduction

Argillaceous siltstone is a weak and water-sensitive geomaterial commonly developed in red-bed formations [1]. Its heterogeneous structure, consisting of framework grains, clay-bearing components, and soluble or weak cementing materials, is susceptible to deterioration under repeated moisture variations [1,2]. Water–rock interaction can weaken interparticle bonding, promote particle rearrangement and crack development, and progressively reduce the strength and stiffness of the rock [2]. At the slope scale, such deterioration may accumulate under rainfall infiltration and groundwater fluctuations, thereby increasing the susceptibility of weak rock layers to deformation and reactivation. Field investigations and previous studies of rainfall- and groundwater-affected landslides have shown that preferential seepage pathways and progressive weakening of weak-rock layers are important controls on long-term slope instability [3,4,5]. Therefore, clarifying the deterioration behavior of argillaceous siltstone under repeated wet–dry cycles is important for both material-scale damage assessment and the engineering evaluation of red-bed slopes.

Extensive experimental studies have established that repeated wetting and drying can substantially alter the mechanical performance of weak and clay-bearing rocks. Early durability investigations showed that the degree of deterioration depends not only on the number of cycles but also on mineral composition, rock fabric, and initial material integrity [6]. Subsequent tests on shaly sandstone, medium-grained sandstone, and clay-bearing sandstone generally reported reductions in compressive or tensile strength, elastic modulus, and resistance to deformation, although the magnitude and rate of these changes varied considerably among different lithologies and testing conditions [7,8,9]. For argillaceous siltstone, multiscale experiments further demonstrated that the degradation of macroscopic mechanical properties is accompanied by changes in particle bonding, pore structure, and fracture development [10]. More recent studies on red-bed rocks have linked this progressive weakening to the accumulation of internal damage and have developed experimental, theoretical, and numerical descriptions of its dependence on wet–dry cycle number [11,12]. Collectively, these studies identify progressive mechanical deterioration as the predominant long-term response. However, because most investigations emphasize the net loss of mechanical properties after a specified number of cycles, temporary or staged changes in strength have rarely been examined in relation to the simultaneous evolution of stiffness, energy partition, strain localization, and failure morphology.

To identify the physical basis of such mechanical deterioration, recent studies have increasingly integrated macroscopic loading tests with microstructural observations, pore characterization, crack monitoring, and energy analysis. Multiscale investigations have demonstrated that wet–dry cycling involves the coupled evolution of mineral–water interactions, intergranular bonding, pore structure, and macroscopic deformation, rather than a simple reduction in strength alone [13,14]. Microscopic observations further show that repeated moisture variations promote particle debonding, surface disintegration, and the initiation and connection of pores and microcracks [15,16]. At the specimen scale, full-field deformation measurements and direct failure observations have revealed that crack development may gradually evolve from localized fracture to multiple interacting damage regions as cycling proceeds [17]. Energy-based analyses provide complementary evidence: the weakening of the load-bearing skeleton generally reduces elastic energy-storage capacity, while particle rearrangement, frictional sliding, and crack propagation consume an increasing proportion of the external work [18]. Recent macro–mesoscopic investigations have further confirmed the close correspondence between pore-network deterioration and the loss of macroscopic mechanical performance under wet–dry cycling [19]. These findings demonstrate that wet–dry deterioration is governed by interacting processes across several observational scales. Nevertheless, microstructural, energy, strain-field, and failure-mode results are frequently interpreted in parallel rather than as a connected causal sequence, limiting their ability to explain complex mechanical responses that depart from continuous monotonic weakening.

Despite these advances, the mechanism linking component-scale alteration to a non-monotonic mechanical response remains insufficiently resolved. Recent multiscale and field investigations have demonstrated that the dissolution of cementing minerals can weaken intergranular bonding, modify pore networks, and contribute to the progressive destabilization of red-bed rocks and slopes [20,21]. However, the formation and enlargement of pores may not be the only consequences of water–rock interaction in clay-rich argillaceous siltstone. Fine particles released from weakened cemented contacts, together with water-sensitive clay-bearing materials, may migrate and locally occupy pre-existing or newly formed voids. Such redistribution may be associated with temporary changes in intergranular contact and frictional resistance without restoring the stiffness and integrity of the original mineral skeleton. Repeated swelling and shrinkage may subsequently destabilize these locally accumulated materials, reopen the occupied pores, and promote distributed cracking and surface spalling. Existing studies have not adequately determined whether such temporary pore occupation is associated with UCS stabilization during intermediate cycles, how this stabilization occurs despite continued deterioration of the original load-bearing skeleton, or how the subsequent loss of the filling materials is reflected in energy partition, strain localization, failure morphology, and microstructural evolution. A unified explanation connecting these processes across different observational scales therefore remains lacking.

To address these issues, purplish-red argillaceous siltstone collected from the Nashuixi ancient landslide was examined in the natural state and after 1, 3, 5, 7, and 9 wet–dry cycles. Uniaxial compression testing was integrated with digital image correlation and strain-energy analysis to characterize the evolution of strength, stiffness, energy storage and dissipation, surface strain localization, and macroscopic failure mode. Scanning electron microscopy, quantitative face-porosity measurements, and water-absorption tests were used to track the accompanying changes in pore morphology and surface damage. X-ray diffraction and X-ray fluorescence analyses conducted at representative cycling stages provided complementary evidence of changes in mineral and elemental composition. The specific objectives were to (1) quantify the non-monotonic mechanical and energy responses during wet–dry cycling; (2) establish the correspondence among energy partition, strain-field evolution, and observed failure patterns; and (3) determine how pore-scale material redistribution and subsequent microstructural damage contribute to the staged mechanical response. The novelty of this study lies in interpreting the intermediate-stage departure from continuous strength loss by jointly considering the decoupled evolution of strength and stiffness, strain-energy partition, DIC-derived strain localization, and mineralogical and pore-structure changes. This multiscale framework helps distinguish temporary apparent strengthening from genuine recovery of the load-bearing skeleton and provides a material-scale basis for evaluating the long-term deterioration of clay-rich weak-rock slopes subjected to repeated rainfall infiltration and groundwater fluctuations.

## 2. Materials and Methods

### 2.1. Geological Setting and Sample Collection

The argillaceous siltstone investigated in this study was collected from the Nashuixi (NSX) ancient landslide in Lichuan, Hubei Province, China, at approximately 108°42′16″ E and 30°12′00″ N. The study area is located within the southwestern Hubei fold belt and is characterized by tectonically dissected and karstified low-to-middle mountainous terrain, with natural slope angles of approximately 15–30°. The exposed bedrock consists predominantly of purplish-red argillaceous siltstone from the middle member of the Triassic Badong Formation (T_2_b_2_) [3]. The strata have dip directions of 310–320° and dip angles of 30–60°. Two dominant joint sets, with attitudes of 54°∠70° and 90°∠70°, provide preferential pathways for rainfall infiltration and groundwater migration [3].

The argillaceous siltstone forms an important weak and water-sensitive layer within the landslide. The landslide has an estimated volume of approximately 2.847 × 10^6^ m^3^ and a principal sliding direction of approximately 153°. It was reactivated on 2 July 2024 following prolonged rainfall during May and June 2024. Field investigations indicated that rainfall infiltration, groundwater accumulation, tectonic discontinuities, and karst-related seepage pathways jointly contributed to its reactivation [3]. This geological setting provides direct engineering context for investigating the progressive deterioration of the argillaceous siltstone under repeated moisture variations.

**Figure 1:**
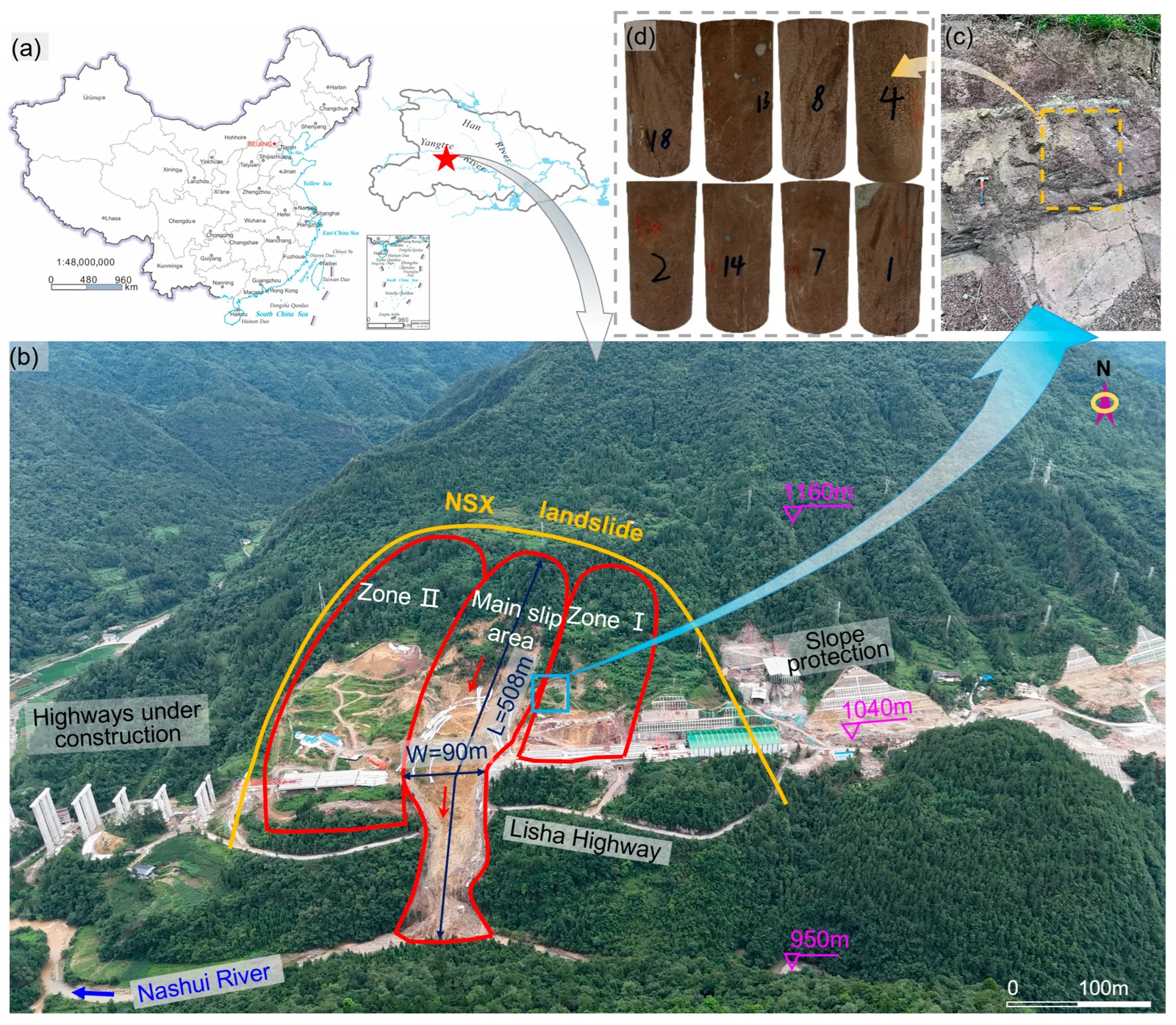
Geological setting and sample collection at the Nashuixi ancient landslide: (**a**) location of the study area; (**b**) aerial view of the Nashuixi (NSX) ancient landslide, in which the yellow line delineates the overall ancient-landslide boundary and the red lines delineate the main slip area, Influence Zone I, and Influence Zone II; (**c**) field exposure and sampling location corresponding to the cyan box in panel (**b**); and (**d**) prepared argillaceous siltstone specimens [3].

According to the previous field investigation [3], the NSX landslide comprises the main slip area, Influence Zone I, and Influence Zone II, as shown in Figure 1b. The main slip area is the narrow central sector that underwent sliding during the 2 July 2024 reactivation event, whereas Zones I and II are the flanking influence sectors that were affected by deformation but did not undergo the main sliding movement. Initial deformation occurred in Zone I following excavation at the slope toe, whereas the subsequent deformation expansion stage involved crack development at the trailing edges of the main slip area and both influence zones. Zone I did not undergo sliding during the event because anti-slide piles had previously been installed at its front edge [3].

Fresh and relatively intact rock blocks were collected from exposed T_2_b_2_ argillaceous siltstone at the landslide site. Immediately after sampling, the blocks were wrapped in multiple layers of plastic film and stored in sealed containers to minimize moisture loss and additional weathering during transportation and storage. The location of the study area, landslide morphology, sampling exposure, and prepared specimens are shown in Figure 1.

### 2.2. Specimen Preparation and Wet–Dry Cycling

The collected rock blocks were cored, cut, and ground into standard cylindrical specimens in accordance with the ISRM suggested method [22]. The specimens had a nominal diameter of 50 mm and a height of approximately 100 mm. The non-parallelism between the two end surfaces was controlled within 0.05 mm, and the perpendicularity deviation between each end surface and the specimen axis was less than 0.25°.

Before wet–dry cycling, each specimen was visually inspected, and its P-wave velocity was measured solely as part of a non-destructive screening procedure to assess initial integrity and homogeneity, rather than to determine dynamic elastic parameters. Specimens without visible defects and with comparable P-wave velocities were selected for the subsequent tests. The selected specimens were assigned to six groups corresponding to the natural state and after 1, 3, 5, 7, and 9 wet–dry cycles, with three specimens used for the uniaxial compression test in each group. Specimens without wet–dry treatment were designated as the natural-state group.

The cycling procedure was established with reference to previous studies on argillaceous siltstone and red sandstone [1,10,16]. Each wet–dry cycle consisted of successive drying, cooling, and wetting stages. During the drying stage, the specimens were placed in a forced-convection oven at 65 °C for 12 h and were subsequently transferred to a desiccator to cool to room temperature. During the wetting stage, the cooled specimens were completely immersed in pure water at room temperature for 12 h. After immersion, each specimen was removed, and free water on its surface was gently wiped off. One drying stage followed by one wetting stage was defined as one complete wet–dry cycle.

After each wetting stage, the specimen mass was measured using an electronic balance with an accuracy of 0.01 g. Water absorption, *w*, was calculated as follows [1,10,16]:

(1) $$w=\frac{m_{w}-m_{d}}{m_{d}}\times 100\%$$

where *m_w_* is the specimen mass measured after immersion and removal of surface water, and *m_d_* is the corresponding dry mass measured before immersion.

### 2.3. Mechanical Testing, DIC, and Energy Analysis

Specimens in the natural-state group were tested without wet–dry treatment, whereas specimens in the cycling groups were tested after the prescribed final wetting stage and removal of free surface water. Because the natural-state reference group and the cycling groups were tested under different moisture conditions, the difference between the natural-state and 1-cycle groups cannot be attributed exclusively to the first wet–dry cycle and may partly reflect the influence of the different moisture conditions during testing. Uniaxial compression tests were conducted using a DS-1000 rigid testing machine (Sichuan Dexiang Kechuang Instrument Co., Ltd., Chengdu, China) under displacement-controlled loading at a rate of 0.05 mm/min. Load and axial displacement were continuously recorded until complete specimen failure. Axial stress was calculated using the initial cross-sectional area, and axial strain was determined from the axial displacement and initial specimen height. The unconfined compressive strength (UCS), obtained from the uniaxial compression test, was defined as the peak axial stress. The secant elastic modulus, E_50_, was calculated from the slope of the line connecting the origin of the stress–strain curve to the point corresponding to 50% of the peak stress on the ascending branch. The UCS results were summarized as mean ± standard deviation (SD). The difference between the 1- and 5-cycle groups was evaluated using a two-sided Welch’s *t*-test, with *p* < 0.05 considered statistically significant.

Digital image correlation (DIC) has been widely used to characterize full-field deformation, strain localization, and crack development in rock specimens under loading [23,24]. Accordingly, DIC was employed to establish correspondence among the mechanical response, energy evolution, and surface deformation of the specimens. Before loading, one longitudinal surface of each specimen was coated with a uniform background layer and a random, high-contrast speckle pattern. The camera was positioned perpendicular to the observation surface, and stable and uniform illumination was maintained throughout the test. Images were continuously acquired and synchronized with the mechanical loading data.

**Figure 2:**
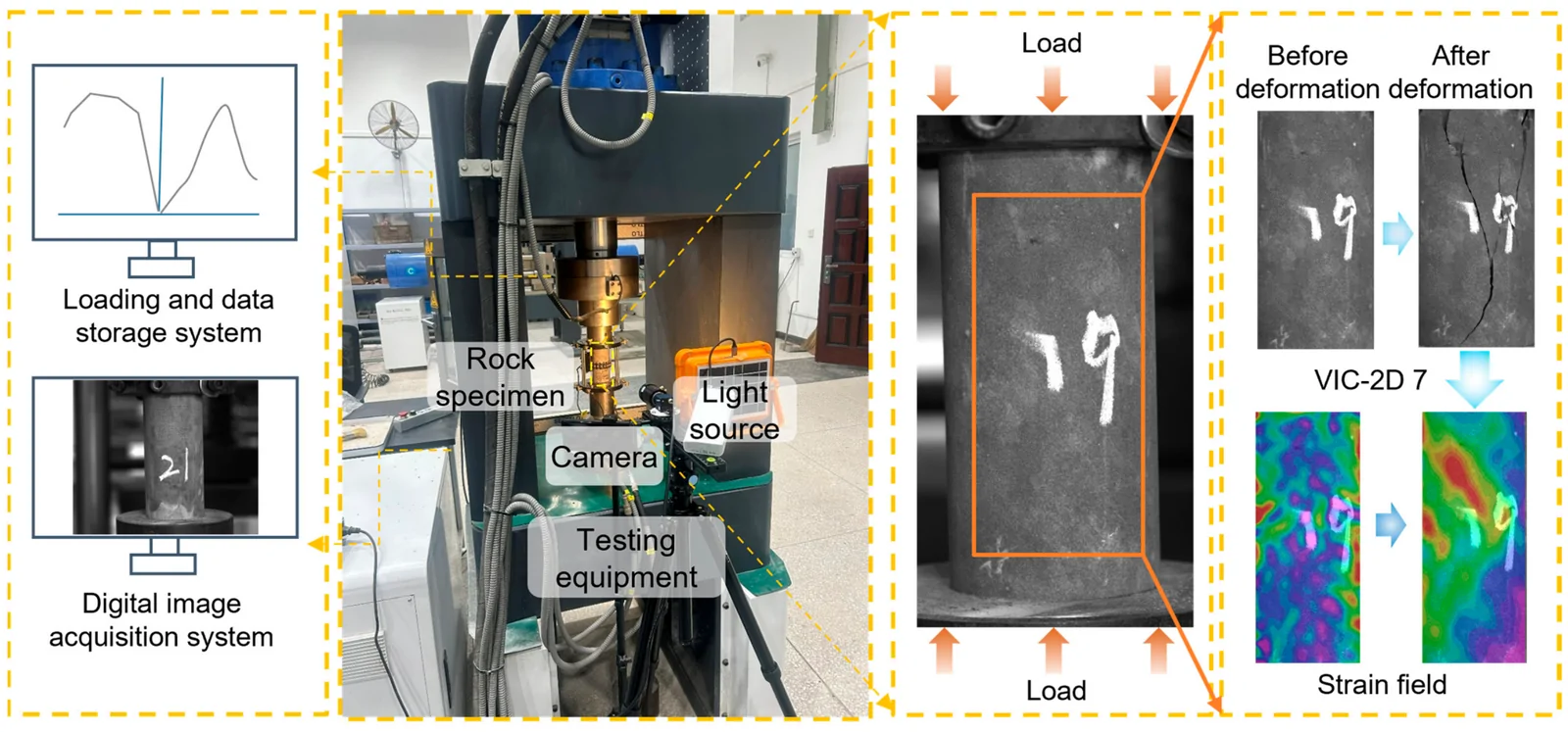
Experimental setup for synchronous uniaxial compression testing and digital image correlation image acquisition.

The acquired image sequences were processed using VIC-2D 7 to obtain full-field surface strain distributions. Characteristic stress points, denoted as *σ_A_–σ_E_*, were identified from changes in the stress–strain and energy evolution curves. Images corresponding to these loading states were extracted to examine the initiation, propagation, and coalescence of localized deformation. The synchronized loading and image-acquisition system is shown in Figure 2.

The energy calculation followed the method adopted by Du et al. [25] and established energy-partition approaches for dry, saturated, cyclically loaded, and wet–dry-treated sandstone [26,27,28]. Under quasi-static uniaxial loading, heat exchange between the specimen and its surroundings was neglected. The external work applied to the specimen was converted into total strain energy density, *U*, consisting of elastic strain energy density, *U_e_*, and dissipated energy density, *U_d_* [26]:

(2) $$U\;=\;U_{e}\;+\;U_{d}$$

At a selected loading point *i*, the total strain energy density was calculated from the area under the axial stress–strain curve:

(3) $$U_{i}=\int_{0}^{\varepsilon_{i}}\sigma d\varepsilon$$

where *σ* and *ε* are the axial stress and axial strain, respectively, and *ε_i_* is the axial strain at the selected loading point.

The elastic strain energy density was approximated as

(4) $$U_{e,i}=\frac{1}{2}\sigma_{i}\varepsilon_{e,i}\approx \frac{\sigma_{i}^{2}}{2E_{50}}$$

where *σ_i_* is the axial stress at the selected loading point, *ε_e,i_* is the corresponding recoverable elastic strain, and *E*_50_ is the elastic modulus used for energy calculation. This approximation treats the stress–strain response as equivalent linear elasticity characterized by E_50_. Because wet–dry cycling increases the nonlinearity of the material response, the calculated elastic strain energy should be regarded as an approximate measure for comparison among groups rather than an exact measure of recoverable energy. The dissipated energy density was then obtained as

(5) $$U_{d,i}=U_{i}-U_{e,i}$$

The dissipated energy ratio, *η_d,i_*, was calculated as

(6) $$\eta_{d,i}=\frac{U_{d,i}}{U_{i}}\times 100\%$$

At peak stress, *σ_i_* was taken as the UCS, and the corresponding values of *U*, *U_e_*, *U_d_*, and *η_d_* were used to compare the energy storage and dissipation characteristics among the different wet–dry cycling groups. The total strain energy represents the external work absorbed by the specimen, the elastic strain energy represents the recoverable energy stored within the load-bearing skeleton, and the dissipated energy represents irreversible energy consumption associated with particle rearrangement, intergranular friction, and crack development.

### 2.4. Microstructural and Compositional Characterization

Following the mechanical tests, representative blocks measuring approximately 5 mm × 5 mm × 3 mm were collected from fresh fracture surfaces. The blocks were dried and coated with an approximately 10 nm thick gold layer to minimize surface charging. Microstructural observations were conducted using an SU8010 field-emission scanning electron microscope (Hitachi High-Technologies Corporation, Tokyo, Japan) operated at an accelerating voltage of 5.0 kV and a working distance of 8.0 mm. For qualitative multiscale observations, images at magnifications of 50×, 500×, 1000×, and 2000× were acquired from the same selected specimen within each cycling group to examine surface disintegration, pore filling, crack propagation, and material detachment at different observational scales. Because the specimens were examined after compression, individual microcracks and detached particles could not be attributed exclusively to wet–dry cycling. The SEM results were therefore interpreted comparatively among groups subjected to identical mechanical testing and preparation procedures.

**Figure 3:**
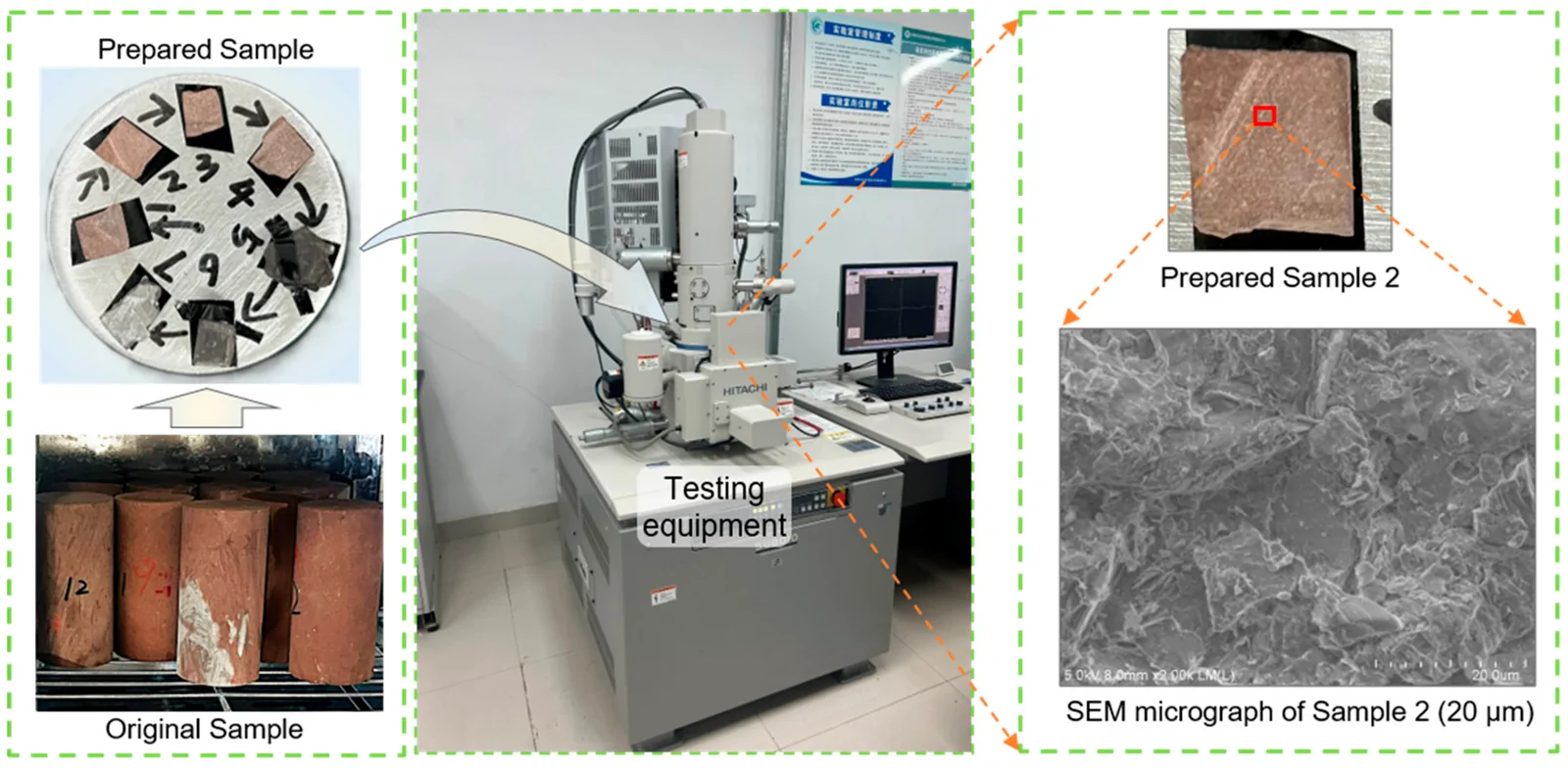
Experimental workflow for scanning electron microscopy observation and face-porosity extraction.

The two-dimensional face porosity was extracted from the SEM images using ImageJ (https://imagej.net/ij/, accessed on 16 July 2024). Each image was first processed using median filtering to reduce high-frequency noise and was then converted into a binary image through threshold segmentation. Pore pixels were separated from the solid matrix, and the face porosity, *P_f_*, was calculated as

(7) $$P_{f}=\frac{A_{p}}{A_{t}}\times 100\%$$

where *A_p_* is the total area occupied by pore pixels and *A_t_* is the total image area. For each cycling group, four non-overlapping SEM fields were selected at 500× magnification from spatially separated regions of the same selected specimen to reduce excessive concentration of the observations within a single local area. The fields were chosen from well-focused surface areas without obvious imaging artifacts or edge effects, and the same field-selection and image-processing criteria were applied to all cycling groups. The four fields represent subsampling within a single specimen rather than independent specimen-level replicates. Their arithmetic mean was therefore reported as the mean face porosity of the selected specimen. Although this consistent procedure enables comparative description among groups, the resulting values characterize only selected two-dimensional surface fields and may not capture the full spatial heterogeneity or three-dimensional pore structure of the specimens. The procedure for specimen preparation, image acquisition, and face-porosity extraction is illustrated in Figure 3.

Compositional characterization was conducted on specimens from the natural-state, 5-cycle, and 9-cycle groups, corresponding to the initial condition, intermediate stabilization stage, and subsequent deterioration stage, respectively. X-ray diffraction analysis was performed using a SmartLab X-ray diffractometer (Rigaku Corporation, Tokyo, Japan) with Cu Kα radiation in accordance with the industrial standard SY/T 5163-2018 [29]. Representative material from each selected group was ground to pass through a 0.075 mm sieve, dried, homogenized, and pressed into sample holders. The principal mineral phases were identified from their characteristic diffraction peaks, and their relative contents were quantified using the K-value method specified in SY/T 5163-2018 [29].

X-ray fluorescence spectrometry was conducted on material from the same groups. The results were expressed as normalized elemental contents of Ca, Si, Al, Fe, K, and Mg and were interpreted together with the XRD results to support the assessment of compositional changes associated with wet–dry cycling.

## 3. Results

### 3.1. Evolution of Unconfined Compressive Strength

**Figure 4:**
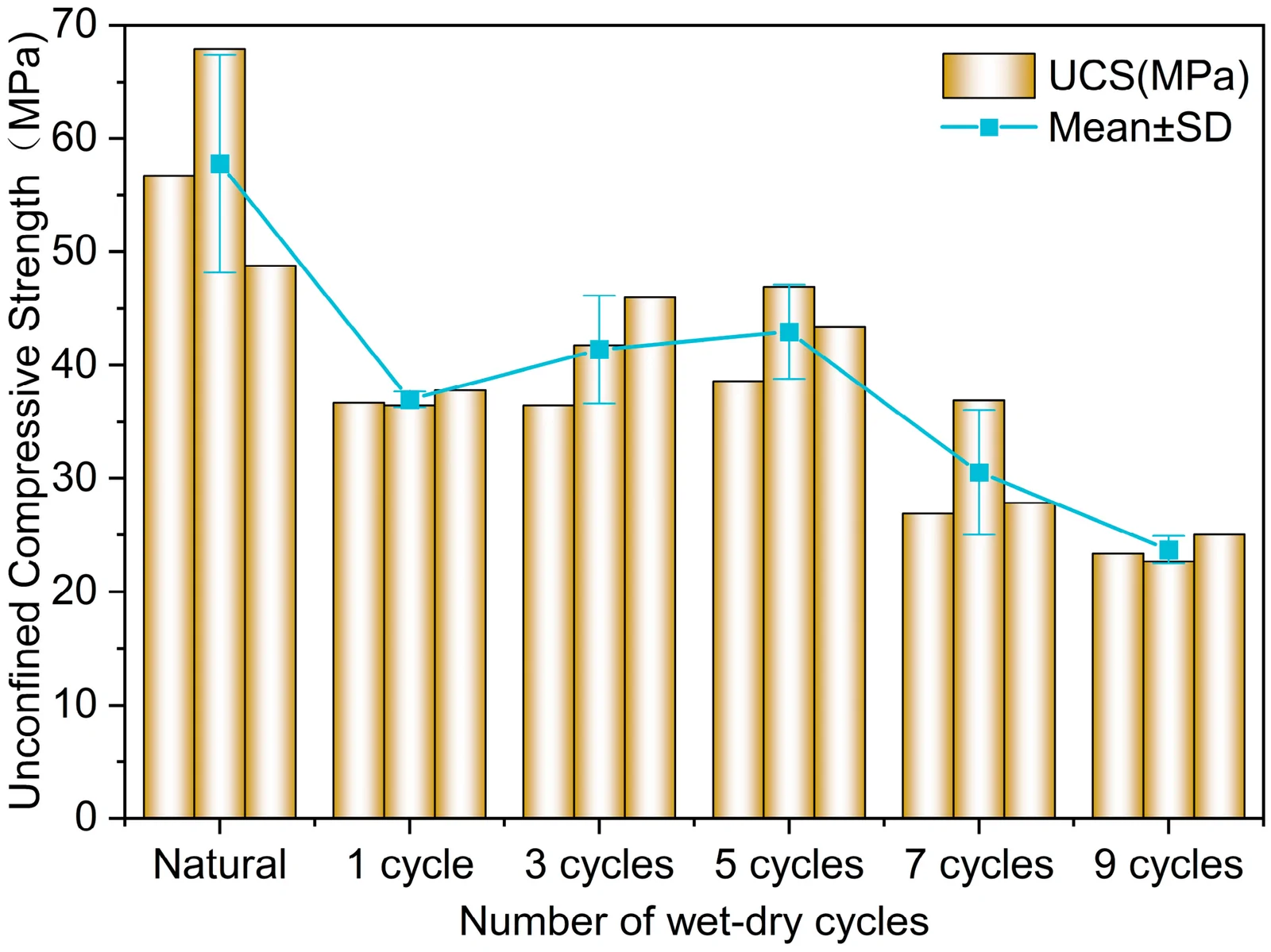
Unconfined compressive strength of argillaceous siltstone after different numbers of wet–dry cycles. Individual test results and mean ± SD (*n* = 3) are shown for each group.

Figure 4 summarizes the unconfined compressive strength results and the corresponding mean values for the different wet–dry cycling groups. The natural-state specimens exhibited a mean UCS of 57.79 MPa. After 1 wet–dry cycle, the mean UCS decreased sharply to 36.99 MPa, representing a reduction of 36.0% relative to the natural-state value.

Following the initial decrease, the mean UCS values were 36.99, 41.40, and 42.96 MPa after 1, 3, and 5 cycles, respectively. Although the mean values showed a modest upward fluctuation, the overlapping standard-deviation ranges suggest that these differences should be interpreted cautiously rather than as clear evidence of strength recovery. The difference between the 1- and 5-cycle groups was not statistically significant according to a two-sided Welch’s *t*-test (*p* = 0.128). Therefore, the UCS response from 1 to 5 cycles is better characterized as an intermediate stabilization stage with modest fluctuations. Throughout this stage, the mean UCS remained 25.7–36.0% lower than the natural-state value.

After 5 cycles, the UCS decreased again, reaching 30.54 MPa after 7 cycles and 23.71 MPa after 9 cycles. The final value was 59.0% lower than that of the natural-state specimens. The mean UCS trend was therefore characterized by an initial sharp decrease, an intermediate stabilization stage with modest fluctuations, and a subsequent renewed decrease.

### 3.2. Mechanical, Energy, and DIC Responses at Selected Cycling Stages

**Figure 5:**
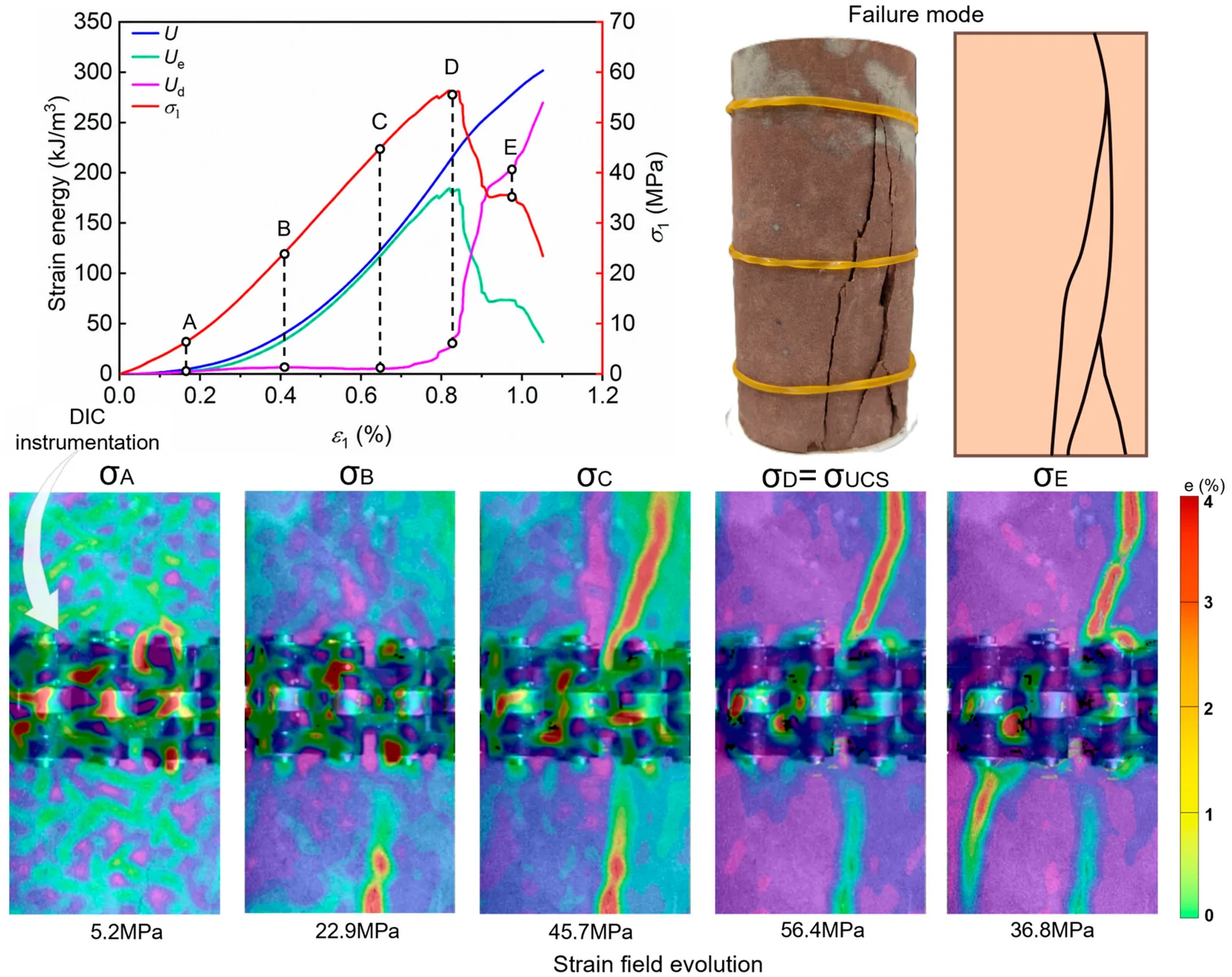
Stress–strain and energy evolution, DIC strain-field distributions, and observed failure mode of the selected natural-state specimen under uniaxial compression.

**Figure 6:**
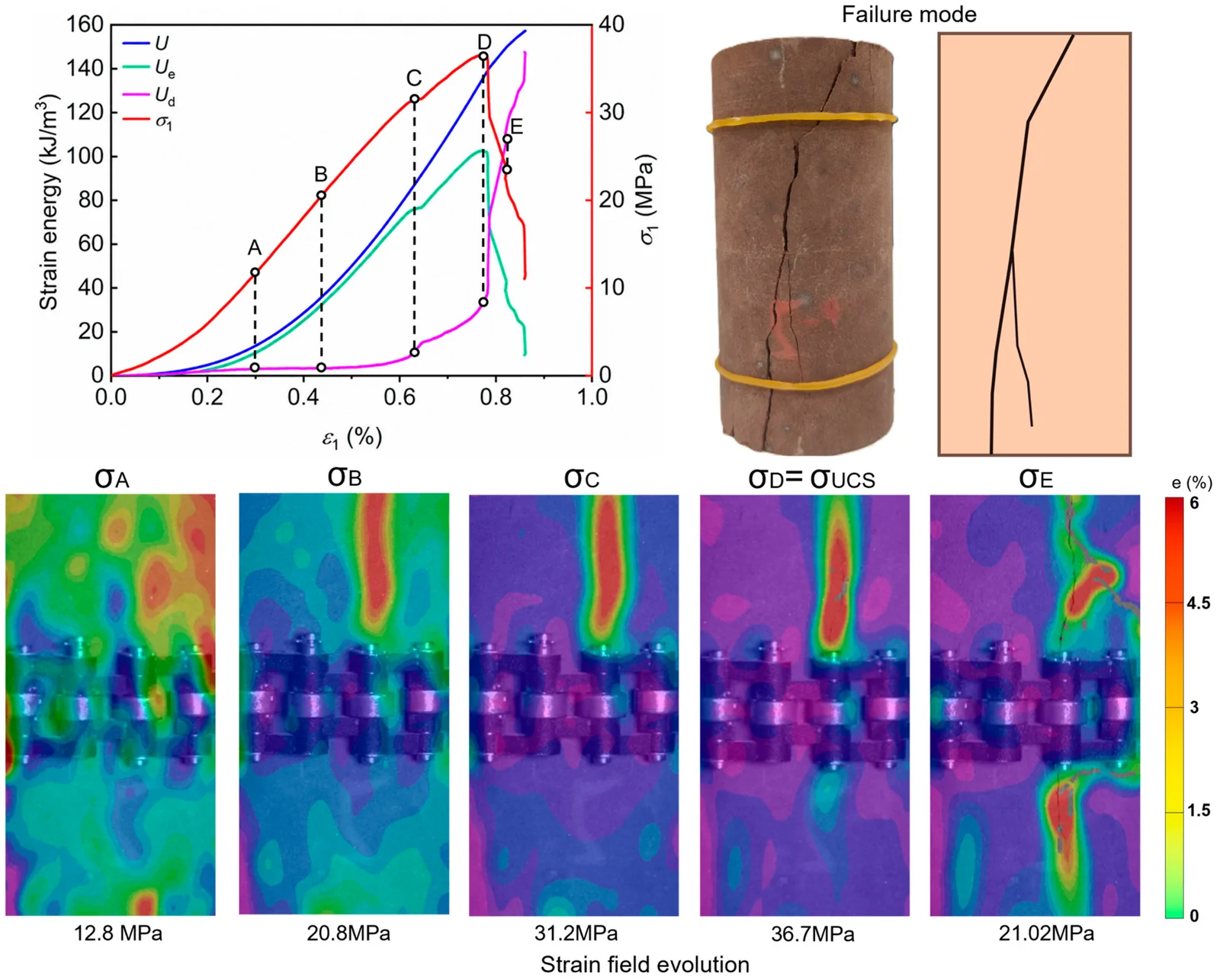
Stress–strain and energy evolution, DIC strain-field distributions, and observed failure mode of the selected specimen after 1 wet–dry cycle under uniaxial compression.

**Figure 7:**
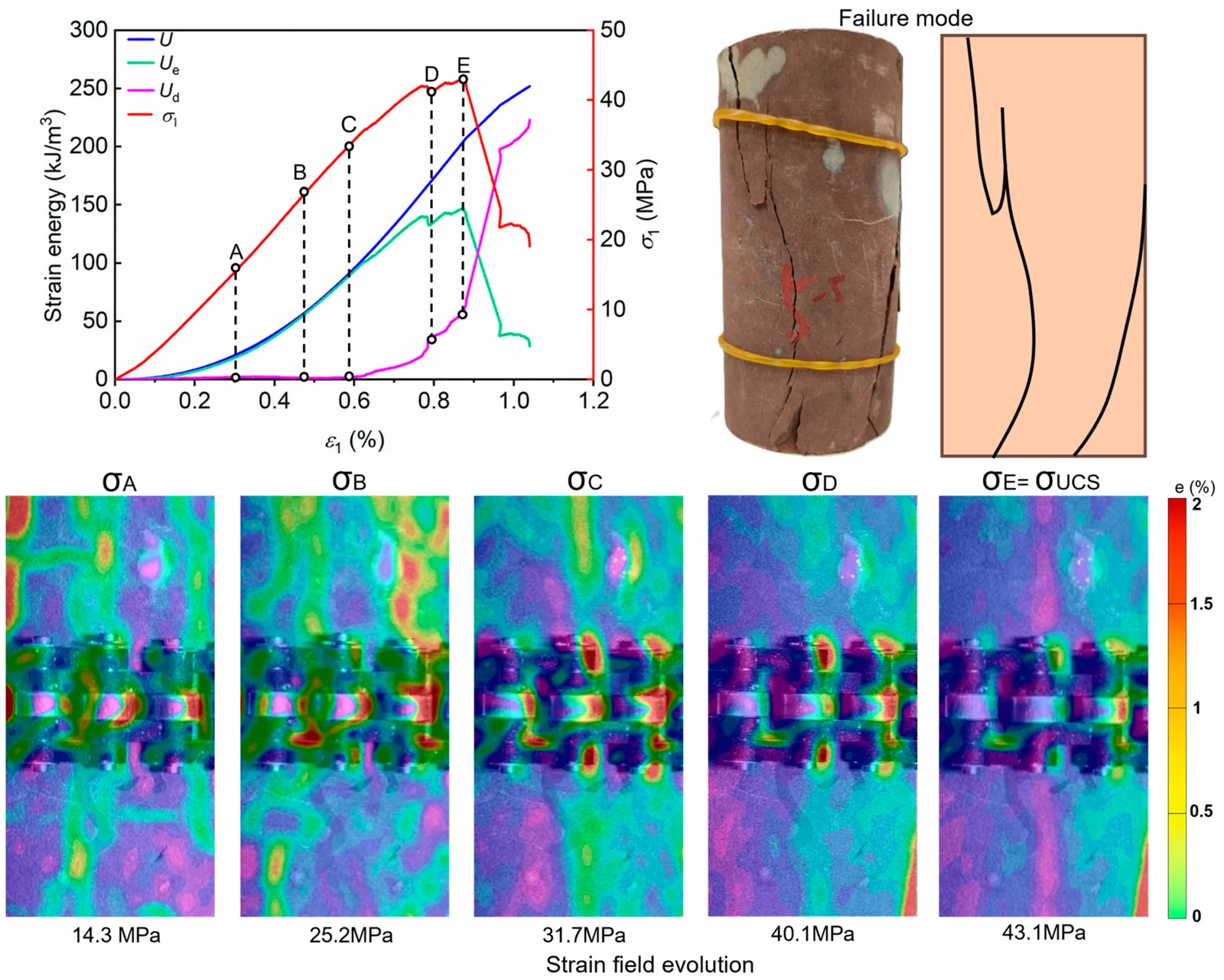
Stress–strain and energy evolution, DIC strain-field distributions, and observed failure mode of the selected specimen after 5 wet–dry cycles under uniaxial compression.

**Figure 8:**
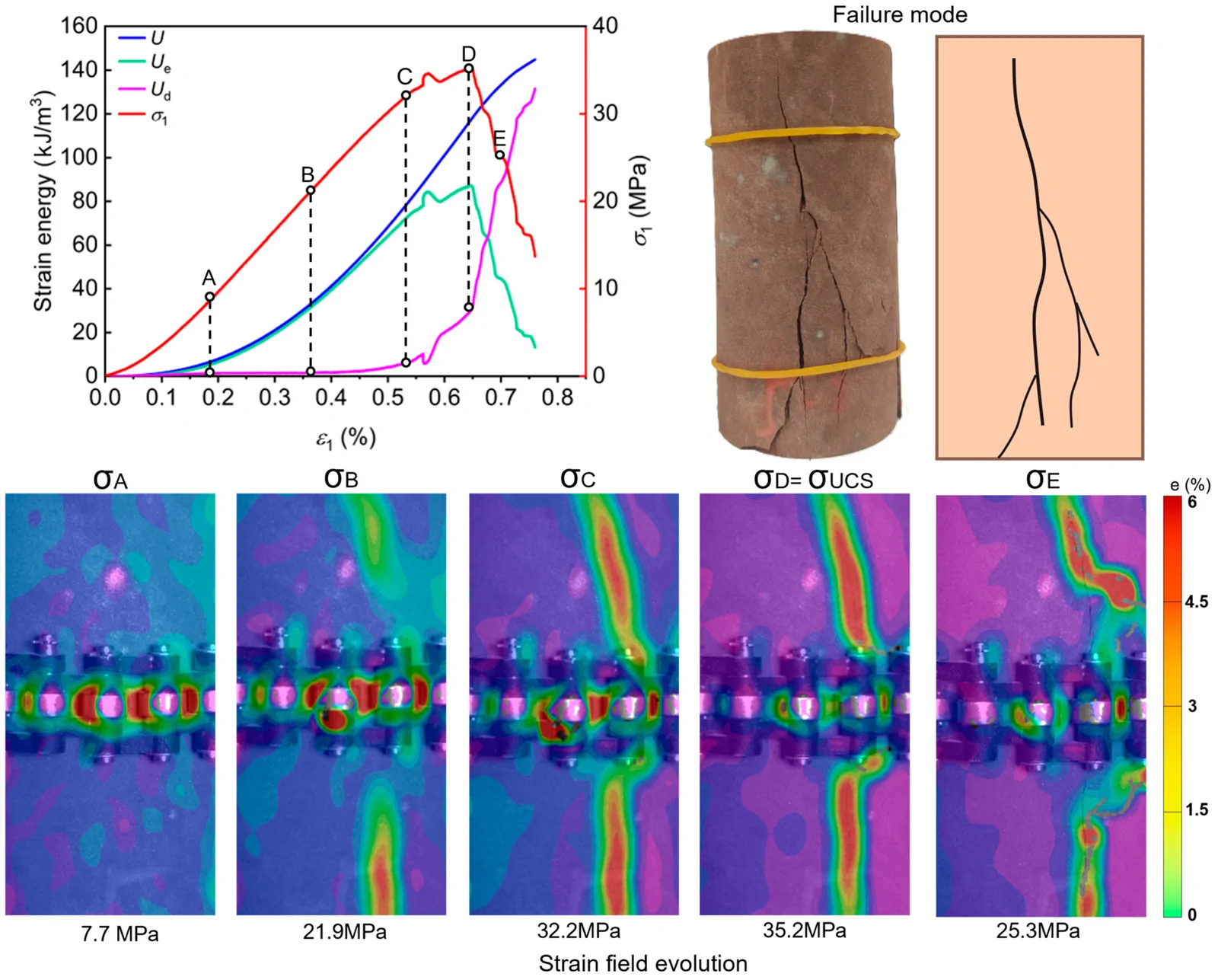
Stress–strain and energy evolution, DIC strain-field distributions, and observed failure mode of the selected specimen after 7 wet–dry cycles under uniaxial compression.

**Figure 9:**
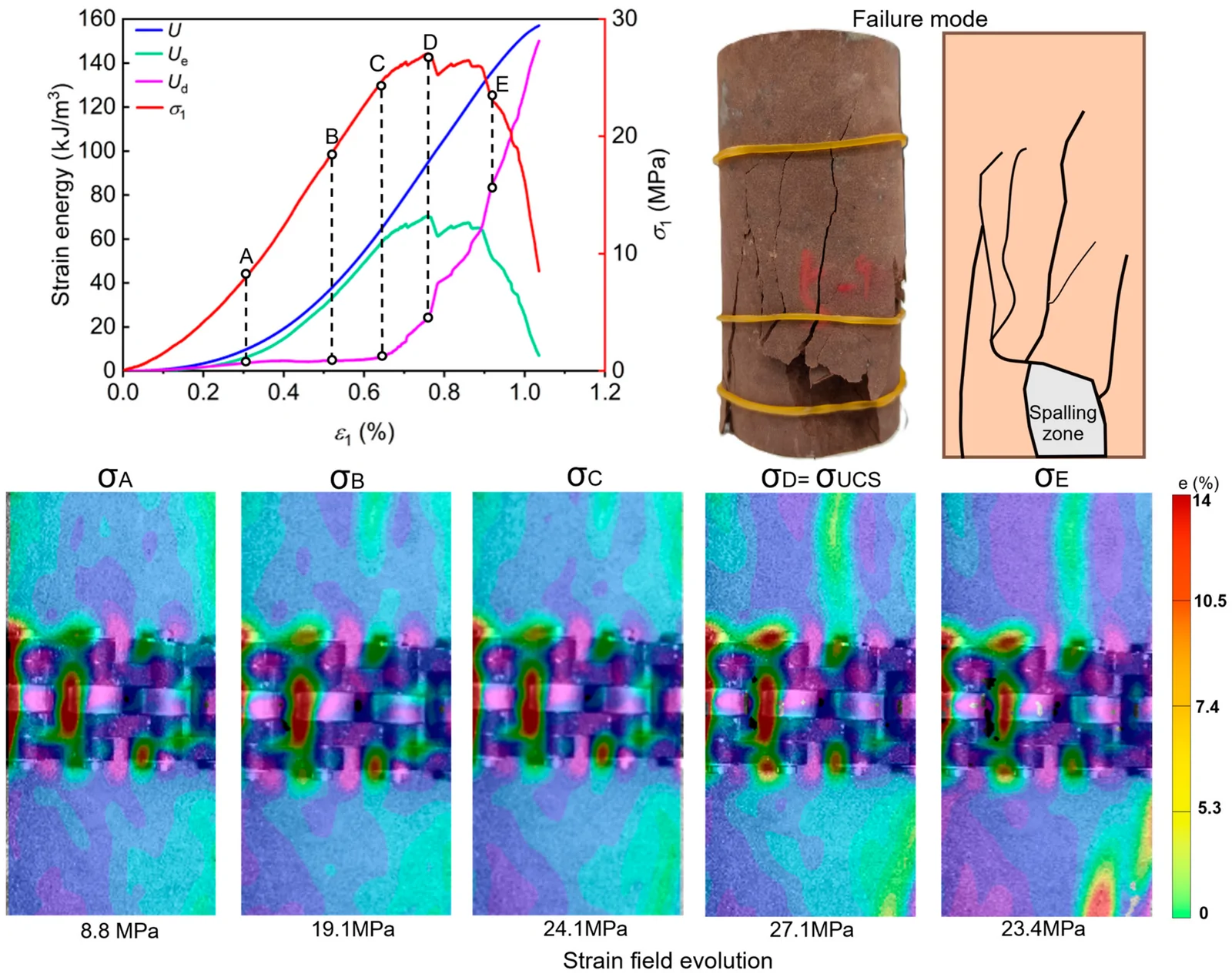
Stress–strain and energy evolution, DIC strain-field distributions, and observed failure mode of the selected specimen after 9 wet–dry cycles under uniaxial compression.

One specimen with a complete stress–strain record and DIC image sequence was selected for detailed comparison at each of the selected cycling stages. The UCS values of the selected specimens were 56.40, 36.70, 43.06, 35.18, and 27.06 MPa for the natural-state, 1-, 5-, 7-, and 9-cycle stages, respectively, compared with the corresponding group means of 57.79 ± 9.63, 36.99 ± 0.69, 42.96 ± 4.17, 30.54 ± 5.53, and 23.71 ± 1.21 MPa (mean ± SD, *n* = 3). The selected specimens from the natural-state, 1-, 5-, and 7-cycle groups fell within the corresponding mean ± SD ranges, whereas the selected 9-cycle specimen exhibited a higher UCS than the group mean ± SD range. Therefore, these single-specimen DIC and energy results are used as illustrative observations of the stage-dependent responses rather than as statistical representations of the group-level behavior. Group-level repeatability and variability are evaluated from the three UCS measurements in each group, as shown in Figure 4. Figure 5, Figure 6, Figure 7, Figure 8 and Figure 9 present the energy evolution, strain-field development, and failure characteristics of these specimens. In Figure 5, Figure 6, Figure 7, Figure 8 and Figure 9, labels A–E denote the selected characteristic loading points corresponding to the DIC strain-field images.

#### 3.2.1. Natural State

The selected natural-state specimen exhibited a UCS of 56.4 MPa and an E_50_ of 8.40 GPa. At peak stress, the total strain energy density was 211.40 kJ/m^3^, of which 184.78 kJ/m^3^ was stored as elastic strain energy and 26.62 kJ/m^3^ was dissipated. The corresponding dissipated energy ratio was 12.59%, indicating that elastic energy storage was predominant before peak failure.

The DIC results show that the surface strain remained relatively limited during the initial loading stages. As the stress approached the peak at *σ_D_*, strain gradually concentrated within a narrow, approximately axial localization band. The band became more pronounced during the post-peak stage, and the maximum surface strain reached approximately 4%.

The observed failure mode was dominated by a longitudinal crack that propagated downward and bifurcated in the lower part of the specimen, forming an overall Y-shaped splitting pattern. The specimen retained relatively high integrity after failure, without pronounced fragmentation or surface spalling.

#### 3.2.2. After 1 Wet–Dry Cycle

After 1 wet–dry cycle, the selected specimen exhibited a UCS of 36.7 MPa and an E_50_ of 6.37 GPa. Compared to the natural-state specimen, the peak total strain energy density decreased by 35.8%, from 211.40 to 135.69 kJ/m^3^. The elastic strain energy density decreased to 102.86 kJ/m^3^, whereas the dissipated energy density increased to 32.83 kJ/m^3^. Consequently, the dissipated energy ratio increased markedly from 12.59% to 24.19%.

The DIC strain fields show that a distinct localization band had already developed at *σ_B_* and subsequently extended in an approximately axial direction. As the stress approached its peak, the localization band became increasingly pronounced, and the maximum surface strain reached approximately 6%, exceeding that observed in the natural-state specimen.

The final failure was characterized by an irregular longitudinal crack with a locally tortuous propagation path. Although the specimen exhibited more pronounced localized deformation than in the natural state, it retained its general integrity after failure, without extensive fragmentation or obvious surface spalling.

#### 3.2.3. After 5 Wet–Dry Cycles

After 5 wet–dry cycles, the selected specimen exhibited a modest increase in UCS to 43.06 MPa. In contrast, E_50_ decreased further to 6.19 GPa and remained substantially lower than the natural-state value. The peak total strain energy density increased to 204.11 kJ/m^3^, approaching the value of the natural-state specimen. However, the elastic strain energy density was 147.18 kJ/m^3^, whereas the dissipated energy density increased to 56.93 kJ/m^3^, approximately 2.14 times the natural-state value. The corresponding dissipated energy ratio reached 27.89%, the highest among the selected specimens.

At approximately 50% of the peak stress, the dissipated energy ratio was only 5.18%, indicating that most irreversible energy consumption occurred during the later stages of loading. This stage-dependent energy evolution differed from the modest increase observed in the UCS and peak total strain energy.

The DIC strain fields remained comparatively uniform during most of the loading process. No continuous strain-localization band was observed before peak stress, and the maximum surface strain was approximately 2%, the lowest among the selected cycling stages.

Several visible cracks formed during final failure, but the specimen retained a relatively intact splitting pattern. Extensive fragmentation and pronounced surface spalling were not observed.

#### 3.2.4. After 7 Wet–Dry Cycles

After 7 wet–dry cycles, the UCS of the selected specimen decreased again to 35.18 MPa, while E_50_ was 7.06 GPa and remained below the natural-state value. The peak total strain energy density decreased markedly from 204.11 kJ/m^3^ after 5 cycles to 116.45 kJ/m^3^. The elastic and dissipated energy densities were 87.14 and 29.31 kJ/m^3^, respectively, corresponding to a dissipated energy ratio of 25.17%. Although the absolute dissipated energy density decreased relative to that after 5 cycles, its proportion of the total strain energy remained approximately twice the natural-state value.

The DIC strain fields show that a continuous, approximately axial localization band reappeared during loading and became progressively more pronounced as the stress approached its peak. Additional strain-concentration regions developed around the principal band, indicating a broader spatial distribution of localized deformation than that observed after 5 cycles.

The final failure was characterized by a dominant longitudinal crack accompanied by several oblique branch cracks. Both the number and spatial extent of visible cracks increased relative to the 5-cycle specimen, although pronounced surface spalling was not yet observed.

#### 3.2.5. After 9 Wet–Dry Cycles

After 9 wet–dry cycles, the selected specimen exhibited the lowest UCS and E_50_ among the selected cycling stages, with values of 27.06 MPa and 4.78 GPa, respectively. The peak total strain energy density decreased to 94.21 kJ/m^3^, corresponding to less than half of the natural-state value. The elastic and dissipated energy densities were 70.41 and 23.80 kJ/m^3^, respectively. Despite the decrease in their absolute values, the dissipated energy ratio remained high at 25.27%.

Before peak stress, a small stress drop from 24.1 to 23.4 MPa occurred between *σ_C_* and *σ_D_*. During the same loading interval, several distinct strain-concentration regions developed at spatially separated positions on the specimen surface. These regions expanded and partially coalesced as loading continued. The maximum surface strain reached approximately 14%, substantially exceeding those of the other selected specimens.

The final failure involved multiple intersecting cracks, visible fragment detachment, and a distinct surface-spalling zone near the lower part of the specimen. The observed failure mode therefore differed from the relatively intact splitting patterns of the earlier cycling stages and was characterized by distributed multi-crack failure accompanied by surface spalling.

Overall, the selected specimens exhibited a progressive change in their coupled mechanical, energy, strain-field, and failure responses. The natural-state specimen was dominated by elastic energy storage and localized splitting. After 1 cycle, the energy-storage capacity decreased markedly, while the dissipated energy ratio and degree of strain localization increased. After 5 cycles, the modest increases in UCS and total strain energy occurred without a corresponding recovery in stiffness, and the dissipated energy ratio reached its maximum value. During the subsequent 7- and 9-cycle stages, the total strain energy decreased again, strain concentration developed across multiple regions, and the observed failure pattern evolved from longitudinal splitting with branch cracks to distributed multi-crack failure with surface spalling.

### 3.3. Microstructural and Compositional Evolution

**Figure 10:**
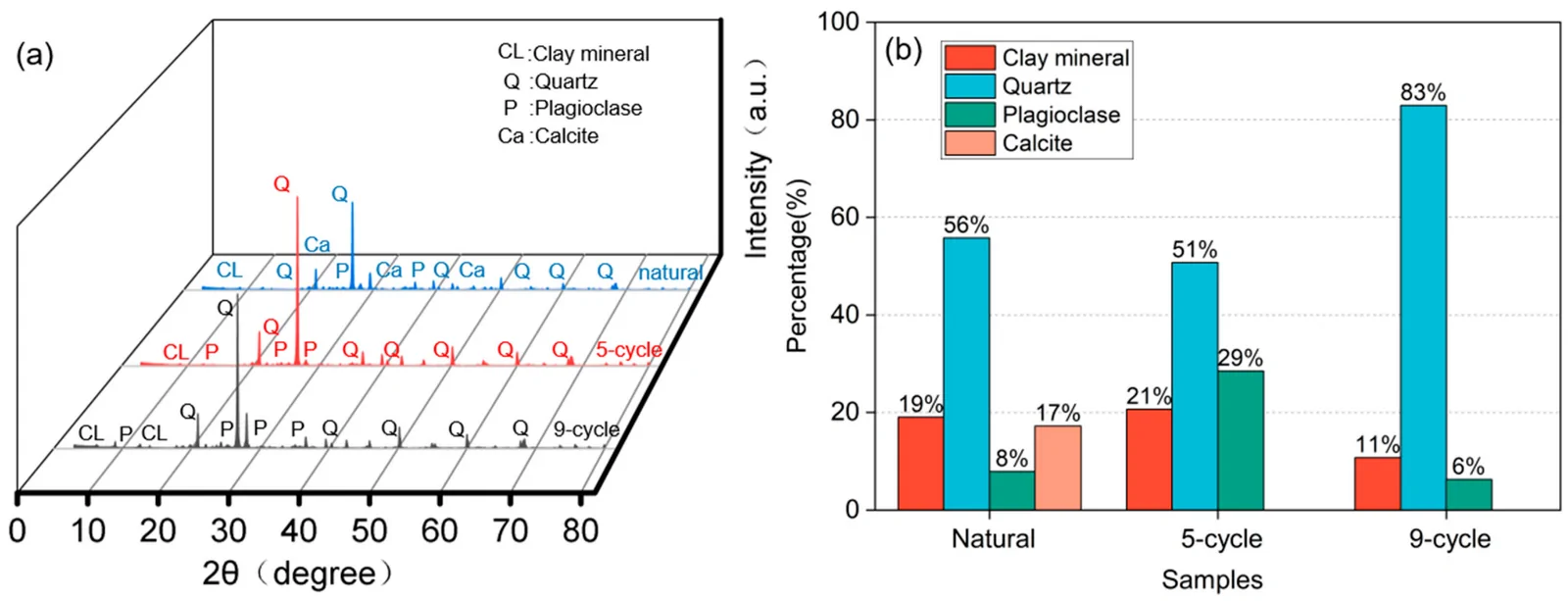
Mineralogical characteristics of argillaceous siltstone in the natural state and after 5 and 9 wet–dry cycles: (**a**) XRD patterns; (**b**) relative contents of the detected mineral phases.

**Figure 11:**
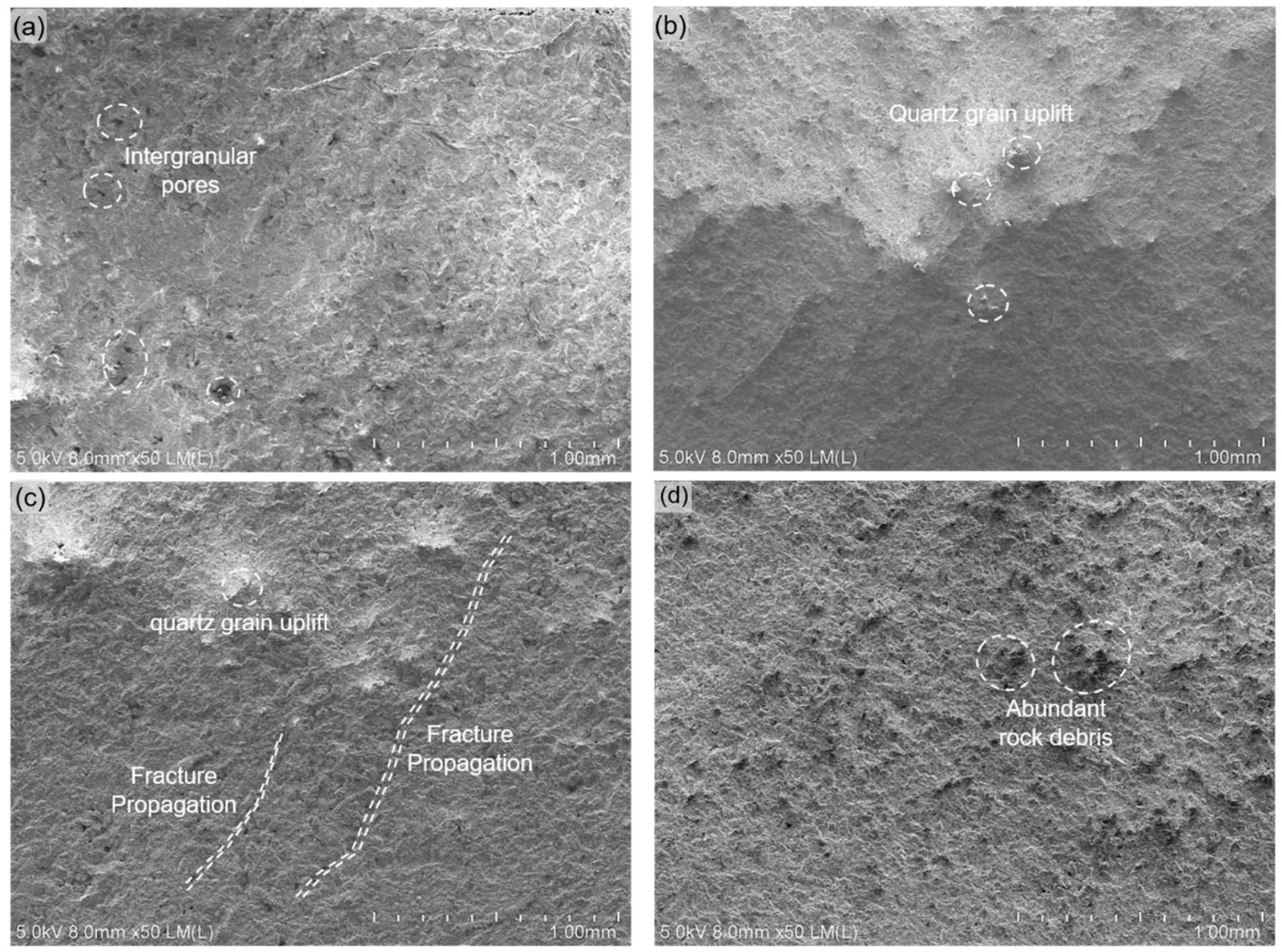
SEM images of argillaceous siltstone at 50× magnification: (**a**) natural state; (**b**) after 5 wet–dry cycles; (**c**) after 7 wet–dry cycles; and (**d**) after 9 wet–dry cycles.

**Figure 12:**
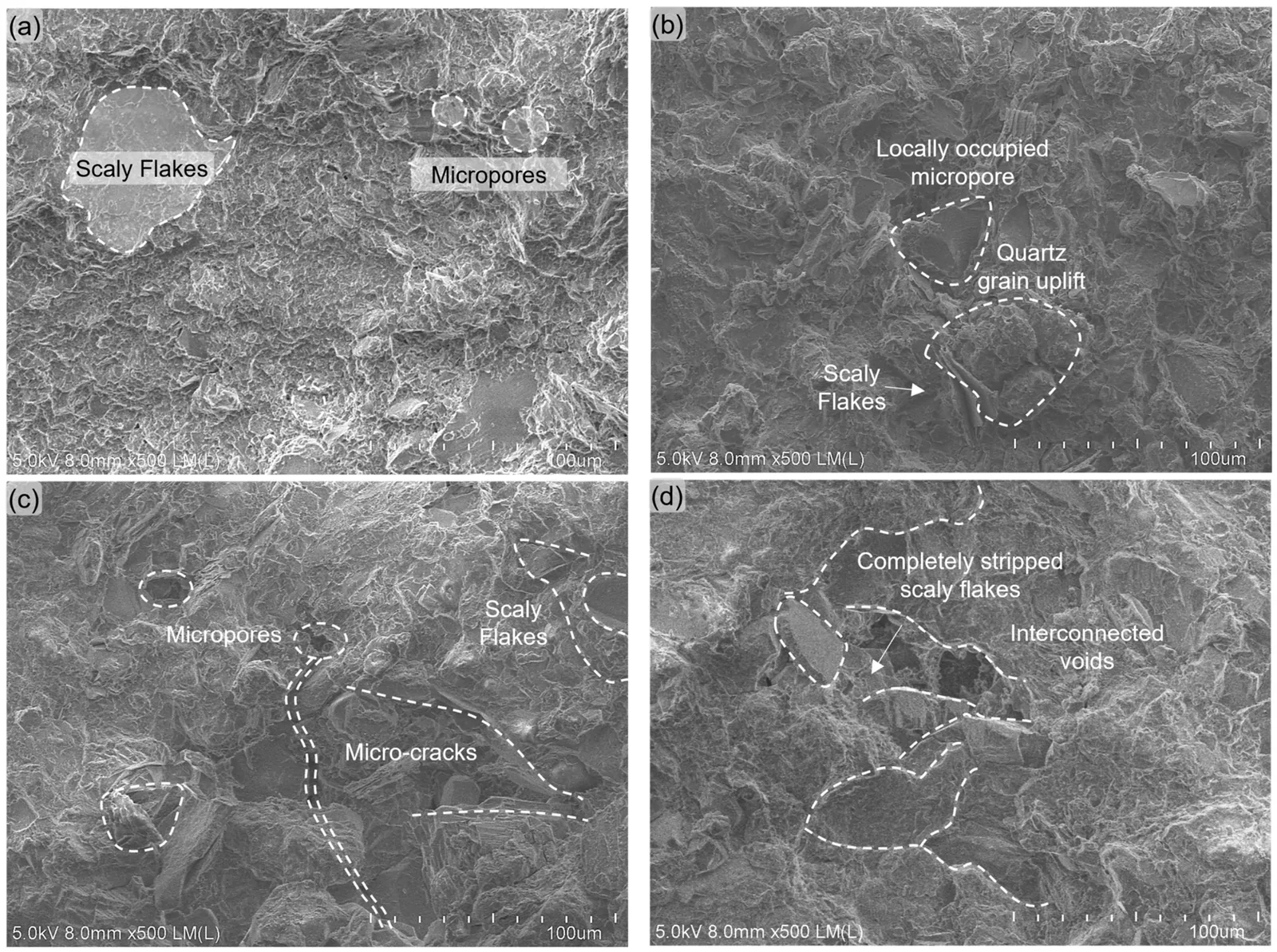
SEM images of argillaceous siltstone at 500× magnification: (**a**) natural state; (**b**) after 5 wet–dry cycles; (**c**) after 7 wet–dry cycles; and (**d**) after 9 wet–dry cycles.

**Figure 13:**
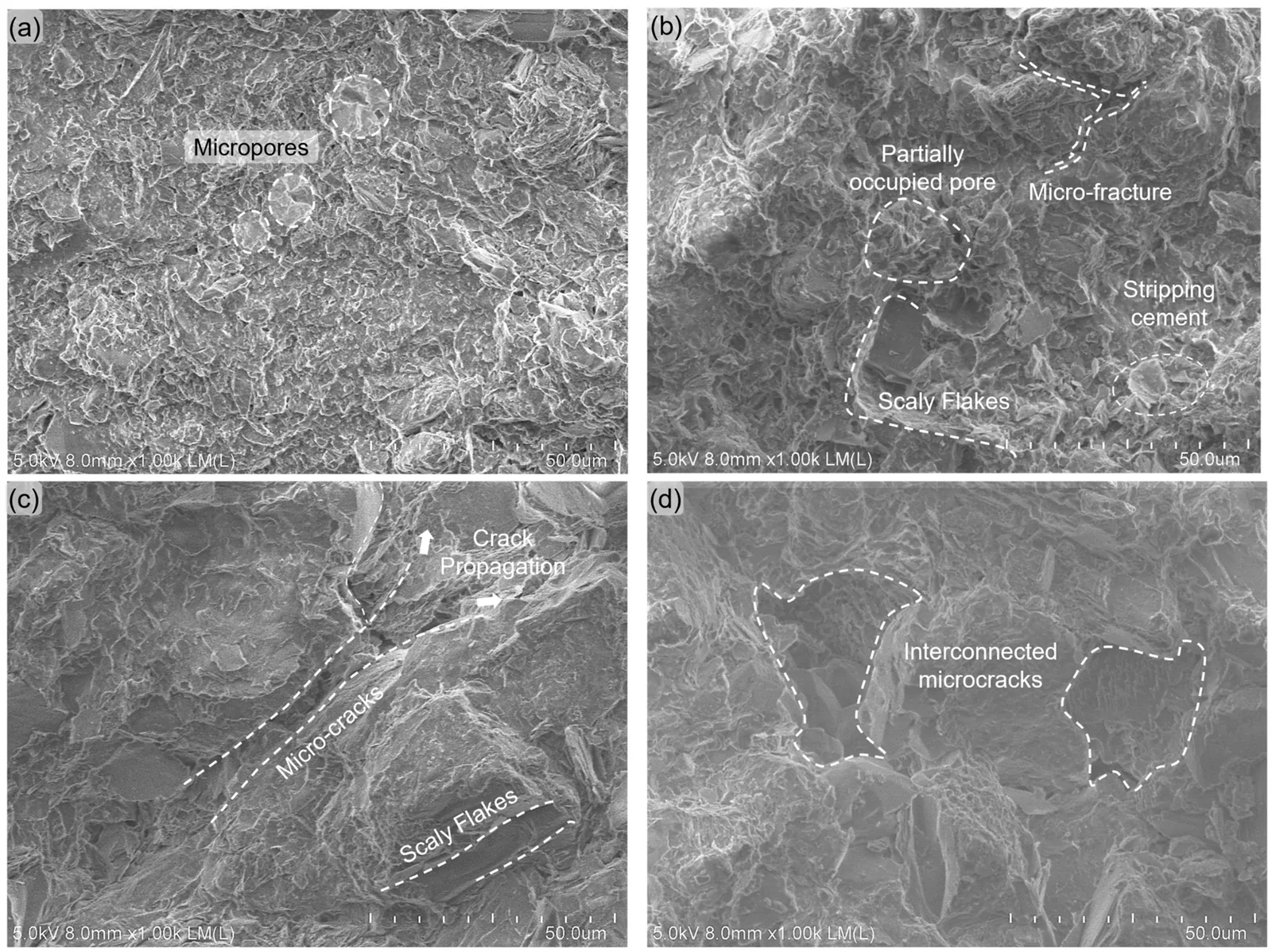
SEM images of argillaceous siltstone at 1000× magnification: (**a**) natural state; (**b**) after 5 wet–dry cycles; (**c**) after 7 wet–dry cycles; and (**d**) after 9 wet–dry cycles.

**Figure 14:**
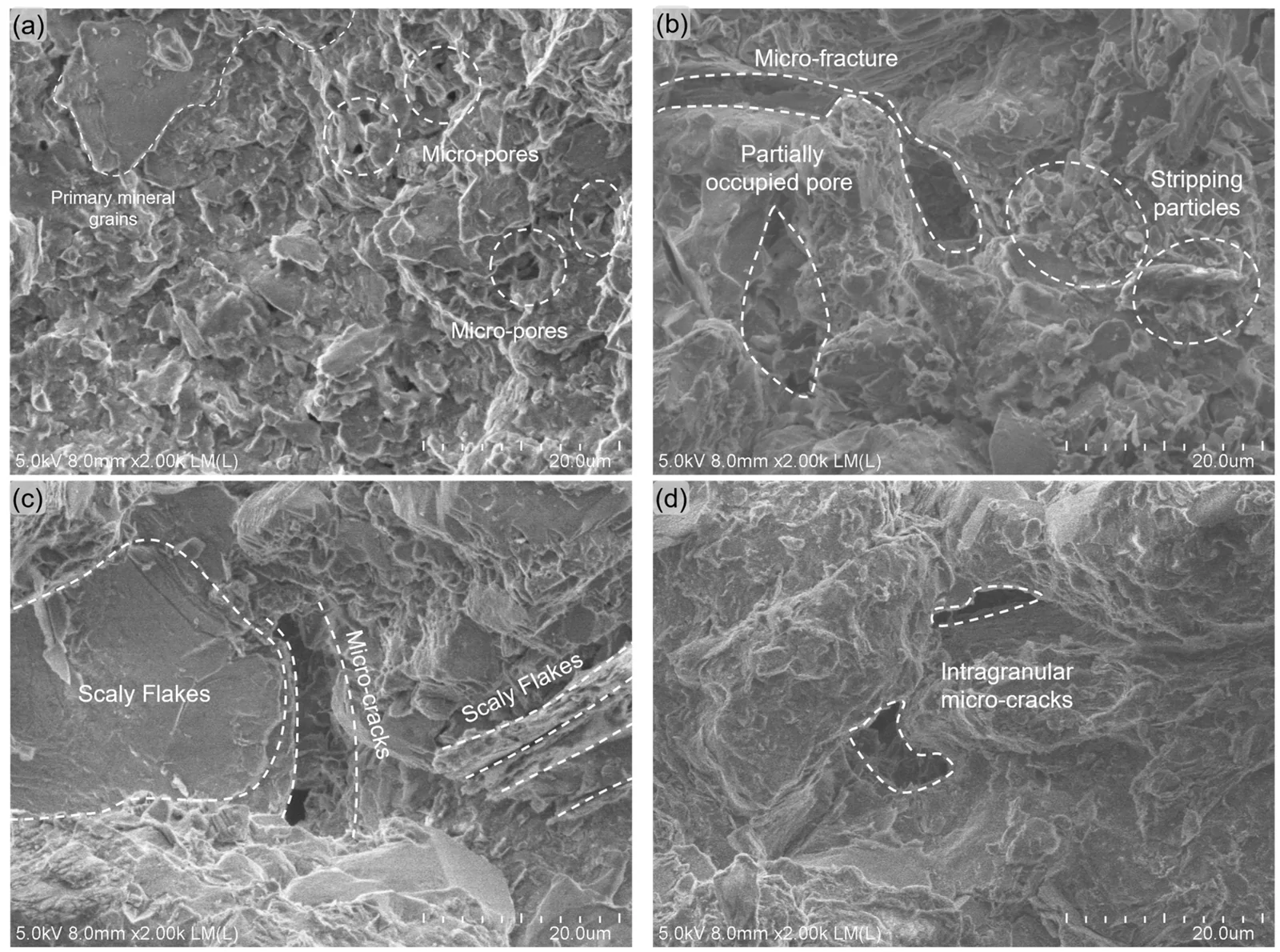
SEM images of argillaceous siltstone at 2000× magnification: (**a**) natural state; (**b**) after 5 wet–dry cycles; (**c**) after 7 wet–dry cycles; and (**d**) after 9 wet–dry cycles.

**Figure 15:**
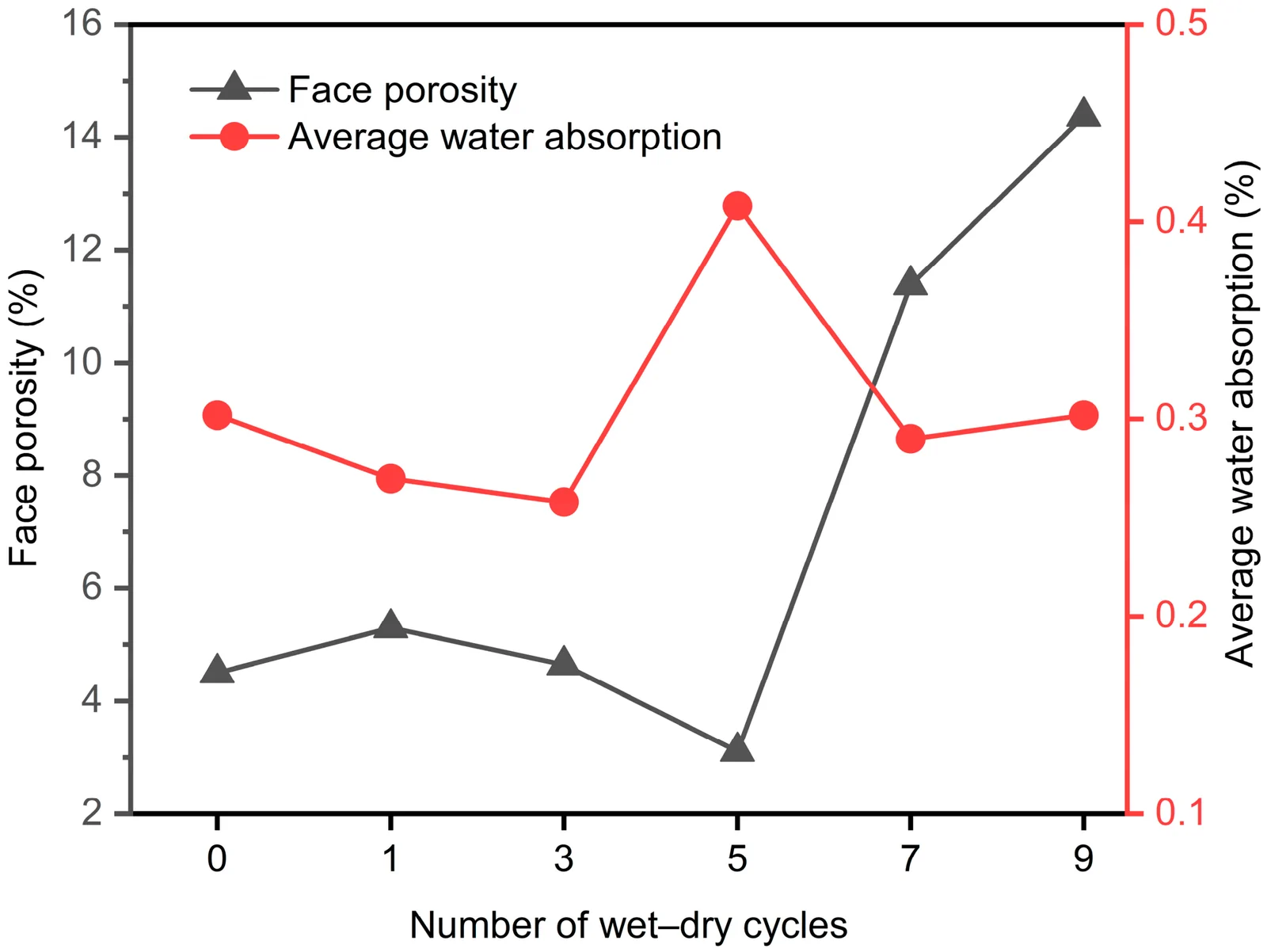
Mean face porosity and average water absorption of argillaceous siltstone after different numbers of wet–dry cycles.

To characterize the material changes accompanying the mechanical, energy, and strain-field responses, XRD and XRF analyses, multi-magnification SEM observations, and quantitative measurements of face porosity and water absorption were conducted. The corresponding results are presented in Figure 10, Figure 11, Figure 12, Figure 13, Figure 14 and Figure 15, and Table 1.

#### 3.3.1. Mineralogical and Elemental Changes

Figure 10 presents the XRD patterns and relative mineral contents of the specimens in the natural state and after 5 and 9 wet–dry cycles. The detected mineral phases consisted primarily of quartz, clay minerals, plagioclase, and calcite. In the natural-state specimen, calcite accounted for 17% of the detected mineral composition, and its characteristic diffraction peak was observed at 2θ ≈ 29.5°. This peak was not detected in the specimens after 5 and 9 cycles, indicating a pronounced reduction in the detectable calcite phase.

The remaining mineral phases exhibited staged changes in their relative proportions. The relative quartz content changed from 56% in the natural state to 51% after 5 cycles and subsequently increased to 83% after 9 cycles. The relative clay-mineral content changed from 19% to 21% and then decreased to 11%, whereas the relative plagioclase content changed from 8% to 29% and subsequently decreased to 6%. These results show that the pronounced reduction in detectable calcite was accompanied by non-monotonic variations in the relative proportions of the remaining mineral phases. The reported mineral contents are semi-quantitative values normalized to the detected crystalline phases; therefore, their variations are interpreted as relative rather than absolute changes in mineral abundance.

The XRF results are summarized in Table 1. Among the selected elements, Ca exhibited the most pronounced change, decreasing from 7.24% in the natural state to 0.34% after 5 cycles and remaining at a low level of 0.59% after 9 cycles. The normalized contents of Si, Al, Fe, K, and Mg showed comparatively moderate and non-monotonic variations. The substantial decrease in Ca was consistent with the non-detection of the calcite diffraction peak after wet–dry cycling.

#### 3.3.2. Microstructural Evolution Observed by SEM

Figure 11 presents the microstructural characteristics observed at 50× magnification. The natural-state specimen exhibited a relatively compact and intact surface, with only a limited number of visible intergranular pores. After 5 cycles, localized separation appeared along the edges of several mineral grains, while some intergranular materials showed signs of loosening and deformation. After 7 cycles, visible microcracks extended along preferential directions across the observed surface. After 9 cycles, loose debris became widely distributed, and the cracks exhibited a more irregular and dispersed spatial pattern.

At 500× magnification, the natural-state specimen contained relatively intact overlapping platy particles and isolated micropores (Figure 12). After 5 cycles, some micropores were locally occupied by fine-grained materials, while the edges of the platy particles showed slight loosening. After 7 cycles, tortuous microcracks developed along grain boundaries, accompanied by increasingly pronounced edge warping and particle loosening. After 9 cycles, extensive detachment of platy particles exposed distinct surface depressions, and previously isolated pores were accompanied by larger, more connected voids.

Figure 13 shows the microstructural characteristics at 1000× magnification. In the natural state, dispersed micropores were present within the matrix, with relatively smooth pore walls and well-defined boundaries. After 5 cycles, several pores were partially occupied by fine-grained materials, while small gaps and incipient microcracks appeared at grain contacts. After 7 cycles, the microcracks extended along existing interfaces, and the bonding between adjacent platy particles became visibly weakened. After 9 cycles, multiple microcracks intersected and coalesced, forming a more continuous fracture network.

At 2000× magnification, the natural-state specimen exhibited relatively smooth grain surfaces and small intergranular pores (Figure 14). After 5 cycles, short microcracks appeared on several grain surfaces, accompanied by localized debris detachment. After 7 cycles, the platy particles showed more pronounced separation along preferential orientations. After 9 cycles, cracks extended across individual grains in addition to the intergranular cracks, indicating that the observed damage had expanded from grain boundaries to include intragranular cracking.

#### 3.3.3. Evolution of Face Porosity and Water Absorption

Figure 15 compares the mean face porosity obtained from the SEM images with the average water absorption of the specimens subjected to different numbers of wet–dry cycles. In the natural state, the face porosity and average water absorption were 4.50% and 0.30%, respectively. During the first 3 cycles, the face porosity showed only limited fluctuations, with values ranging from 4.64% to 5.30%, while the water absorption decreased slightly.

After 5 cycles, the face porosity decreased to 3.11%, the minimum value among the investigated groups and approximately 31% lower than the natural-state value. In contrast, the water absorption increased to its maximum value of 0.41%, approximately 37% higher than the initial value. Thus, the two parameters exhibited distinctly contrasting changes at this cycling stage.

During the subsequent cycles, the face porosity increased sharply to 11.38% after 7 cycles and 14.37% after 9 cycles. The latter value was approximately 3.2 times the natural-state value. Over the same interval, the water absorption decreased to approximately 0.29% after 7 cycles and then increased slightly to approximately 0.30% after 9 cycles. Therefore, between 7 and 9 cycles, both parameters increased, although the change in water absorption was comparatively small.

Overall, the face porosity exhibited limited early fluctuations, reached its minimum after 5 cycles, and increased rapidly during the later cycling stage. By comparison, the water absorption reached its maximum after 5 cycles and subsequently returned to approximately its initial level.

## 4. Discussion

### 4.1. Mechanism of the Intermediate UCS Stabilization

The UCS of the argillaceous siltstone decreased sharply after the first wet–dry cycle, remained broadly stable with modest fluctuations between 1 and 5 cycles, and subsequently decreased again. The combined mechanical and microstructural results suggest that this non-monotonic response may reflect the competition between progressive weakening of the original load-bearing skeleton and temporary changes in local particle contact.

The initial strength reduction may have been associated with degradation of carbonate cementation. In the natural-state specimen, the normalized Ca content was 7.24%, and calcite accounted for 17.23% of the detected mineral phases. By 5 cycles, the Ca content had decreased to 0.34%, and the characteristic calcite peak was no longer detected. This pronounced compositional change, together with the early decrease in UCS and the SEM-observed loosening of grain contacts, suggests that carbonate-cement degradation may have contributed to the early weakening of the rock structure. Calcite can act as an intergranular cement in sedimentary rocks; its dissolution reduces bonding between framework grains and facilitates the development of pores and microcracks. Comparable relationships among water–rock interaction, carbonate-cement degradation, pore development, and mechanical weakening have been reported for water-sensitive red-bed and carbonate-bearing rocks [20,21,30,31].

The intermediate UCS stabilization was accompanied by a different set of microstructural characteristics. After 5 cycles, the SEM images showed fine-grained materials locally occupying some exposed pores, while the face porosity reached its minimum value of 3.11%. The relative proportion of clay minerals also increased slightly from 19.05% in the natural state to 20.68% after 5 cycles. Taken together, these observations are consistent with the local redistribution of fine debris and clay-bearing materials within the weakened pore structure. During repeated wetting and drying, particles released from loosened grain contacts may migrate over short distances and occupy nearby voids. The simultaneous occurrence of local pore occupation and UCS stabilization suggests an association between particle redistribution and the temporary maintenance of load-bearing capacity. Similar pore filling by fine clay particles generated during water–rock interaction has been reported in sandstone subjected to wet–dry cycles [32], while other studies have shown that local pore-space alteration can modify the hydraulic and mechanical responses of sandstone [33,34].

The minimum face porosity after 5 cycles occurred simultaneously with the maximum water absorption of 0.41%. These observations characterize different aspects of the pore system: face porosity represents the exposed pore area within selected two-dimensional SEM fields, whereas water absorption reflects specimen-scale water uptake. Their contrasting changes suggest that local occupation of exposed pores did not eliminate water-accessible pathways or the water-retention capacity of the specimen. Water-sensitive clay-bearing materials and internal defects could continue to contribute to bulk water uptake even where some surface pores were locally occupied.

The intermediate stabilization therefore did not represent restoration of the original mineral skeleton. Although the UCS increased to 43.06 MPa and the peak total strain energy returned to 204.11 kJ/m^3^ after 5 cycles, E_50_ remained at only 6.19 GPa, substantially below the natural-state value of 8.40 GPa. Meanwhile, the dissipated energy ratio reached 27.89%, compared to 12.59% in the natural state. These results indicate that the specimen temporarily maintained part of its load-bearing and energy-absorption capacity while continuing to undergo considerable particle rearrangement, frictional sliding, and irreversible deformation. The presence of fine-grained materials within the pores may have been associated with changes in local load transfer, but could not reproduce the bonding capacity and stiffness of the original carbonate cement. The intermediate UCS stabilization is therefore interpreted as temporary apparent strengthening rather than genuine structural recovery, consistent with observations that compliant filling materials and pore-space alterations can modify load transfer and energy dissipation without reconstructing the original rock skeleton [25,34].

### 4.2. Coupled Evolution of Energy Dissipation and Multiscale Damage

Energy partition provides a macroscopic measure of how the rock structure responds to external loading. Elastic strain energy represents the recoverable energy stored within the load-bearing skeleton, whereas dissipated energy is associated with irreversible processes such as particle rearrangement, intergranular friction, pore compaction, and crack development [27,28]. Consequently, neither the absolute dissipated energy nor its proportion should be interpreted independently of the total energy absorbed before failure [35,36].

The natural-state specimen stored most of the absorbed external work as elastic strain energy. At peak stress, its total strain energy density was 211.40 kJ/m^3^, of which 184.78 kJ/m^3^ was elastic and only 26.62 kJ/m^3^ was dissipated. The corresponding dissipated energy ratio was 12.59%. After 1 cycle, the total strain energy density decreased by 35.8% to 135.69 kJ/m^3^, whereas the dissipated energy density increased to 32.83 kJ/m^3^ and its proportion rose to 24.19%. This redistribution indicates that the specimen stored less energy elastically and consumed a greater proportion of the absorbed work through irreversible deformation after the initial wet–dry treatment.

A distinctive energy response occurred after 5 cycles. The total strain energy density recovered to 204.11 kJ/m^3^, approaching the natural-state value, but the elastic component remained at only 147.18 kJ/m^3^. In contrast, the dissipated energy density increased to 56.93 kJ/m^3^, approximately 2.14 times the natural-state value, and the dissipated energy ratio reached its maximum of 27.89%. The increase in total strain energy therefore reflected an increased capacity to absorb external work rather than restoration of the original elastic energy-storage capacity. This distinction is consistent with the simultaneous stabilization of UCS, continued reduction in E_50_, and SEM-observed local occupation of pores by fine-grained materials. The observed changes in local particle contact were consistent with the temporary maintenance of the load-bearing response, while rearrangement and friction among the weakly bonded materials continued to consume substantial energy during loading.

During the subsequent cycling stage, both the energy-absorption and elastic-storage capacities decreased again. The total strain energy density decreased to 116.45 kJ/m^3^ after 7 cycles and 94.21 kJ/m^3^ after 9 cycles. The corresponding dissipated energy densities also decreased to 29.31 and 23.80 kJ/m^3^, respectively, but the dissipated energy ratios remained above 25%. The reduction in absolute dissipated energy therefore does not indicate mitigation of internal damage. Instead, the weakened specimens reached failure after absorbing substantially less external work, while irreversible deformation continued to account for a large proportion of the available energy.

The energy changes were accompanied by corresponding microstructural evolution. Between 5 and 9 cycles, the relative clay-mineral content decreased from 20.68% to 10.78%, while the face porosity increased from 3.11% to 14.37%. More importantly, the SEM images directly showed progressive particle loosening, material detachment, renewed exposure of voids, and the transition from grain-boundary cracks to intersecting intergranular and intragranular cracks. Considered together, these observations are consistent with the destabilization and progressive loss of materials that had locally occupied pores during the intermediate cycling stage. Similar correspondences between pore-structure degradation, energy redistribution, and macroscopic weakening have been observed in wet–dry-treated sandstone [18,19,36].

The DIC strain fields recorded the accompanying evolution of surface deformation. In the natural-state and 1-cycle specimens, deformation was mainly concentrated within a dominant localization band. After 5 cycles, the strain field remained comparatively uniform before peak stress, despite the high dissipated energy ratio. After 7 cycles, a continuous localization band reappeared together with additional strain-concentration regions. After 9 cycles, several spatially separated concentration regions expanded and partially coalesced. The strain-field evolution was therefore also non-monotonic: the intermediate stage was characterized by relatively dispersed surface deformation, whereas the later stage involved the development and interaction of multiple localized regions. DIC-based studies have similarly demonstrated that the spatial development of strain localization provides information complementary to conventional stress–strain and energy measurements [23,24].

The observed post-failure patterns provide independent macroscopic evidence of this transition. The natural-state specimen exhibited localized Y-shaped splitting and largely retained its overall integrity. After 1 and 5 cycles, splitting remained predominant, although the number and irregularity of visible cracks increased. After 7 cycles, a dominant longitudinal crack was accompanied by several oblique branches. After 9 cycles, multiple cracks intersected across the specimen surface, accompanied by fragment detachment and a distinct spalling zone. The progressive transition from localized splitting to distributed multi-crack failure is consistent with the SEM-observed expansion of microstructural damage and the persistence of a high dissipated energy ratio.

Overall, the energy response did not evolve synchronously with UCS. The intermediate UCS stabilization occurred together with reduced elastic storage and maximum proportional energy dissipation, whereas the later decrease in absolute dissipated energy resulted from the substantial loss of total energy-absorption capacity. When interpreted jointly, the energy components, SEM observations, DIC strain fields, and post-failure patterns suggest a multiscale sequence from particle-scale loosening and pore alteration to distributed surface deformation and macroscopic failure.

### 4.3. Three-Stage Damage Evolution Mechanism

**Figure 16:**
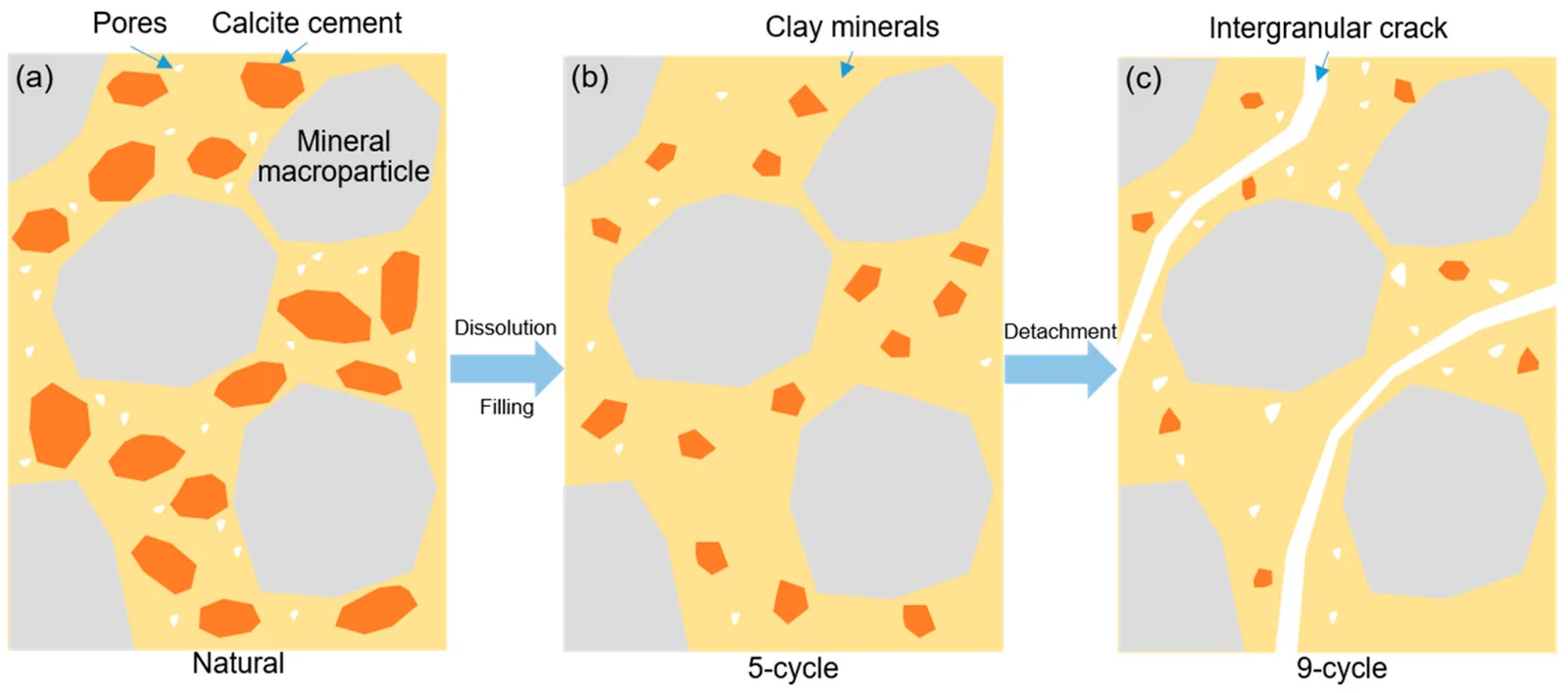
Conceptual three-stage damage evolution mechanism of argillaceous siltstone under wet–dry cycling: (**a**) dissolution-dominated initial stage; (**b**) filling-dominated intermediate stage; and (**c**) detachment-dominated later stage. The stages represent the dominant processes and may partially overlap.

By integrating the mechanical response, energy evolution, DIC strain fields, post-failure patterns, SEM observations, and compositional changes, the deterioration of argillaceous siltstone under wet–dry cycling can be conceptualized as a three-stage dissolution–filling–detachment process (Figure 16). These stages represent changes in the dominant damage process and may partially overlap during cycling. Comparable studies of argillaceous rocks and other chemically degraded porous materials have also demonstrated that compositional alteration, pore-structure evolution, and cracking can interact across different scales [37,38,39].

(a) Dissolution-dominated initial stage. During the initial cycling stage, water entered pre-existing pores and grain contacts and interacted with the cementing materials. By 5 cycles, the normalized Ca content had decreased from 7.24% to 0.34%, while the characteristic calcite diffraction peak was no longer detected. Together with the sharp decrease in UCS after 1 cycle and the SEM-observed loosening of grain contacts, these observations are consistent with the interpretation that degradation and dissolution of carbonate cement may have contributed to the early loss of intergranular bonding. The resulting weakening of cemented contacts facilitated particle separation and the development of additional pore and crack space. Preferential degradation of carbonate-bearing cement and its effects on microstructure and mechanical strength have also been reported in red-bed and carbonate-bearing rocks subjected to water–rock interaction [20,21,31,38].
(b) Filling-dominated intermediate stage. Between 3 and 5 cycles, fine debris released from weakened grain contacts, together with redistributed clay-bearing particles, locally occupied some of the available pores. This interpretation is consistent with the SEM-observed fine-grained materials within pores and the decrease in face porosity to 3.11% after 5 cycles. The simultaneous occurrence of local pore occupation and UCS stabilization suggests an association between particle redistribution and the temporary maintenance of load-bearing capacity. However, the original carbonate cementation was not reconstructed. E_50_ remained substantially lower than the natural-state value, and the dissipated energy ratio reached its maximum of 27.89%, suggesting that particle rearrangement, friction, and irreversible deformation remained active. The intermediate stage was therefore characterized by temporary changes in local load transfer superimposed on continued deterioration of the original mineral skeleton. Similar spatially heterogeneous pore rearrangement and mesostructural changes have been observed in water-affected red-bed rocks [32,33,37].
(c) Detachment-dominated later stage. During the subsequent 7–9 cycles, repeated moisture variations progressively destabilized the fine-grained and platy materials that had locally occupied or covered the pores. SEM images showed particle loosening, material detachment, renewed void exposure, and the coalescence of intergranular and intragranular cracks. Correspondingly, the face porosity increased sharply from 3.11% after 5 cycles to 11.38% after 7 cycles and 14.37% after 9 cycles. The loss of local particle support was accompanied by renewed reductions in UCS and total strain energy. The DIC strain fields developed from a dominant localization band into several interacting strain-concentration regions, while the final failure mode evolved toward distributed multi-crack failure, fragment detachment, and surface spalling. Comparable transitions from particle-scale deterioration to disintegration and distributed failure have been reported for weak mudstone and red-bed argillaceous rocks under wet–dry cycling [40,41,42].

The proposed mechanism provides a plausible explanation for the lack of synchrony between UCS and the other damage indicators. During the intermediate stage, local occupation of pores coincided with changes in local particle contact and the intermediate stabilization of UCS. At the same time, the reduced elastic modulus, elevated dissipated energy ratio, and continued microstructural deterioration suggested that damage to the original skeleton was still accumulating. During subsequent cycling, destabilization and loss of the locally accumulated materials coincided with renewed mechanical weakening and the increasing dominance of damage within the original skeleton. The mechanical response therefore changed from temporary apparent strengthening to renewed strength loss and distributed failure.

Previous wet–dry cycling studies have mainly emphasized progressive strength loss, pore enlargement, crack propagation, and eventual disintegration [7,8,9,10,11,12,13,14,15,16,17,18,19,20,40,41,42], while mineral dissolution and pore-space rearrangement have often been examined as separate processes [20,21,31,32,33,37,38]. Consistent with these studies, the dissolution- and detachment-dominated stages identified here describe early cement degradation and subsequent crack development and particle loss. The present framework further identifies an intermediate filling-dominated stage in which redistributed fine particles locally occupy pores in association with the intermediate stabilization of UCS despite continued stiffness degradation and energy dissipation. By sequentially linking mineral dissolution, temporary pore occupation, and subsequent material detachment with mechanical, energy, DIC, mineralogical, and microstructural evidence, the proposed framework may also be applicable to other clay-rich, weakly cemented rocks subjected to repeated moisture fluctuations.

At the engineering scale, this mechanism provides a material-level interpretation of the progressive weakening of water-sensitive argillaceous siltstone within the Nashuixi landslide. The rock blocks were collected following the major rainfall event associated with landslide reactivation, yet the natural-state material still contained 17.23% calcite. Because no mineralogical data were available before the rainfall event, the extent of calcite loss under field conditions cannot be quantified. Nevertheless, the remaining calcite indicates that field-scale dissolution was incomplete and likely spatially heterogeneous, occurring preferentially along exposed surfaces, fractures, and water-flow pathways. By comparison, the repeated laboratory wet–dry cycles imposed cumulative and controlled water–rock interaction on the prepared specimens, resulting in a much greater reduction in detectable calcite. Although the laboratory cycles do not reproduce the complete field-scale reactivation process, the observed progression from cementation loss and temporary local pore occupation to distributed cracking and surface material loss is consistent with the gradual weakening of the argillaceous siltstone layer under repeated moisture variations [3,20,21]. Consequently, the intermediate stabilization of laboratory UCS should not be interpreted as evidence that the long-term integrity of the corresponding weak-rock layer has been restored.

In slope-stability assessment, the three-stage mechanism can be incorporated by applying moisture-history-dependent reduction factors to the strength and stiffness of argillaceous siltstone weak layers in limit-equilibrium or numerical analyses, following calibration against site-specific tests. Long-term or repeated-rainfall scenarios should consider parameters representative of the later detachment-dominated stage, whereas the intermediate stabilization of UCS should not be treated as recovery of the weak layer. Long-term monitoring should therefore combine hydrological indicators, such as cumulative rainfall, groundwater level, and pore-water pressure, with surface displacement, subsurface deformation, and crack development. A simultaneous increase in hydrological loading and deformation or crack opening may indicate progression toward the detachment-dominated stage and should prompt increased monitoring frequency and renewed slope-stability assessment.

## 5. Conclusions

This study combined mechanical testing, energy analysis, DIC, SEM, face-porosity measurement, XRD, and XRF to clarify the staged deterioration of argillaceous siltstone under wet–dry cycling. The principal conclusions are as follows:

(1) The UCS response was characterized by an initial sharp decrease, an intermediate stabilization stage, and a subsequent renewed decrease. The mean UCS decreased by 36.0% after the first cycle and remained broadly stable, with modest fluctuations, from 1 to 5 cycles before decreasing to 23.71 MPa after 9 cycles. The continued reduction in elastic modulus during the stabilization stage suggests that the temporary maintenance of UCS did not represent restoration of the original load-bearing skeleton.
(2) Energy evolution and full-field deformation revealed damage that could not be identified from UCS alone. After 5 cycles, the dissipated energy ratio reached 27.89%, while the elastic strain energy remained below the natural-state level. With further cycling, the strain fields evolved toward multiple interacting concentration regions, and the failure mode changed from localized splitting to distributed cracking, fragment detachment, and surface spalling. Therefore, the intermediate stabilization of UCS occurred while irreversible damage continued to accumulate.
(3) The combined compositional and microstructural evidence is consistent with a dissolution–filling–detachment mechanism. The reduction in detectable calcite is consistent with the interpretation that degradation of carbonate-cemented grain contacts may have contributed to the early-stage weakening. Fine-grained and clay-bearing materials subsequently occupied some exposed pores, and this local pore occupation coincided with the intermediate UCS stabilization without indicating reconstruction of the original cementation. During later cycles, particle detachment, renewed pore exposure, and interacting intergranular and intragranular cracks became dominant and were accompanied by an increase in face porosity to 14.37% and renewed mechanical deterioration.
(4) These findings suggest that apparently stable short-term UCS does not necessarily indicate stabilization of the internal rock structure. Evaluations of water-sensitive weak-rock slopes should therefore consider stiffness degradation, energy dissipation, strain localization, mineral alteration, and pore-structure evolution together rather than relying solely on compressive strength. Because the laboratory wet–dry cycles simplify field rainfall infiltration, groundwater chemistry, stress conditions, and spatial heterogeneity, further field monitoring and tests under coupled hydrochemical and stress conditions are required to assess the long-term evolution of argillaceous siltstone slopes.

## Figures and Tables

**Table 1:** Normalized elemental contents of argillaceous siltstone in the natural state and after 5 and 9 wet–dry cycles (%).

Group	Si	Al	Fe	Ca	K	Mg
Natural	23.11	7.76	3.33	7.24	1.87	1.88
5-cycle	34.70	6.91	2.51	0.34	1.34	1.45
9-cycle	30.70	8.79	3.49	0.59	2.03	1.95

## Data Availability

The original contributions presented in the study are included in the article, further inquiries can be directed to the corresponding author.
